# Psychometric properties and standardization of the shortened latvian personality inventory (LPI-v3s) in athlete sample: Implications for evidence-based assessment

**DOI:** 10.1371/journal.pone.0352794

**Published:** 2026-07-22

**Authors:** Katrina Volgemute, Viktorija Perepjolkina, Gundega Ulme, Agita Abele, Kristers Ansons, Renars Licis, Rodrigo Lavins, Alina Klonova

**Affiliations:** 1 Department of Health Psychology and Paedagogy, Faculty of Health and Sports Sciences, Riga Stradiņš University, Riga, Latvia; 2 Sports Healthcare Research Center, Riga Stradiņš University, Riga, Latvia; 3 Latvian Academy of Sport Education, Riga Stradiņš University, Riga, Latvia; 4 Department of Neuroscience, Biomedicine, and Movement, University of Verona, Verona, Italy; Soochow University, CHINA

## Abstract

**Background and objective:**

Personality traits are relevant to sport participation and athlete development. Many personality measures used in sport lack systematic psychometric evaluation within athletic populations and are rarely accompanied by population-appropriate normative references. The present research comprised two interlinked studies aimed at empirically examining and refining a shortened version of the Latvian Personality Inventory (LPI-v3s) for use in athlete samples, and at exploring associations between personality traits and sport achievement status.

**Methods:**

A total of 925 athletes (aged 15–45 years) representing 84 sports participated in the study. Following data screening, two subsamples were formed: Study 1 (*n* = 436) for psychometric evaluation and norm development, and Study 2 (*n* = 753) for exploratory criterion-related analyses. In Study 1, exploratory and confirmatory factor analyses were conducted, and measurement invariance across gender was examined using multigroup CFA. Continuous regression-based norming procedures were applied to derive age- and gender-specific T-scores. In Study 2, hierarchical binary logistic regression was used to explore associations between personality traits and competitive status (elite/pre-elite vs. non-elite).

**Results:**

The resulting 53-item LPI-v3s demonstrated an interpretable five-factor structure comprising 14 trait scales, acceptable to good internal consistency (α = 0.72–0.88), and configural, metric, and scalar invariance across gender groups. Exploratory hierarchical binary logistic regression analyses indicated that selected trait-level variables (most notably Anxious Insecurity, Sociability and Orderliness) showed small-to-moderate associations with competitive status, with modest overall classification performance (AUC = 0.64).

**Conclusions:**

The findings support the internal consistency and measurement adequacy of the LPI-v3s within this athlete sample and underscore the importance of contextualized, cautious interpretation of personality scores in sport-related research. Given the provisional nature of the factorial structure and the modest predictive performance observed in Study 2, the LPI-v3s may be useful for research and exploratory assessment contexts involving Latvian-speaking athletes, but should not be used as a diagnostic, selection, or talent-identification tool.

## 1. Introduction

An athlete’s personality shapes patterns of thinking, feeling, and behavior that influence their engagement in sport, psychological functioning, and long-term development [1,2]. Consequently, personality has attracted sustained attention across multiple research domains, including sport psychology. One of the most widely applied theoretical frameworks in this field is the Five-Factor Model [3], which conceptualizes personality in terms of five broad domains: openness, conscientiousness, extraversion, agreeableness, and neuroticism. These dimensions have been linked to a range of psychological characteristics relevant to sport participation, such as stress reactivity, persistence, and self-regulation [4,5].

Empirical research has demonstrated that personality traits vary systematically across gender, competitive level, and type of sport, highlighting substantial heterogeneity within athletic populations [5–7]. At the same time, findings regarding direct associations between specific personality traits and performance outcomes have been inconsistent [2,8]. Rather than indicating a lack of relevance, this variability underscores the contextual sensitivity of personality expression in sport and emphasizes the importance of psychometrically sound, population-appropriate assessment tools capable of capturing stable individual differences across diverse athletic contexts.

### 1.1. Personality assessment in sport

Personality assessment plays an important role in sport psychology by providing structured information about athletes’ psychological characteristics, developmental tendencies, and individual differences that may inform applied practice [9]. In applied settings, personality profiles are commonly used to support individualized psychological interventions, guide coaching strategies, and facilitate communication between athletes and support staff.

Previous research has demonstrated that personality traits vary across competitive levels, sport types, and demographic groups, suggesting that athletic populations are heterogeneous rather than psychologically uniform [5,10]. Certain trait patterns, such as lower levels of neuroticism or higher conscientiousness, have been reported more frequently among high-level athletes in some studies [11,12]. However, these associations are neither universal nor consistent across sports, age groups, or cultural contexts [7].

Importantly, personality traits are not assumed to influence sport outcomes in a direct or deterministic manner. Instead, traits are thought to shape how athletes respond to training demands, competitive stress, and interpersonal environments [13]. For example, lower neuroticism has been associated with greater emotional stability and decision-making efficiency [2,14], while extraversion and conscientiousness have been linked to engagement and persistence in sport-related activities [6]. Sport participation itself may also contribute to adaptive personality development over time, further complicating causal interpretations [15]. These findings highlight that the relevance of personality in sport lies not in identifying universally “optimal” profiles, but in accurately capturing stable individual differences within specific athletic contexts. Personality assessment in sport requires psychometrically sound instruments that are sensitive to sport-specific, demographic, and cultural variation, rather than relying solely on general population measures or global trait assumptions [5,16].

### 1.2. Limitations of existing personality measures in athletic populations

Personality inventories grounded in the Five-Factor Model have been widely applied in sport psychology. While useful, their applicability to sport-specific contexts has been increasingly questioned. Recent research highlights the importance of empirically validating personality measures within athletic populations to ensure accurate and interpretable assessment [17,18]. Studies indicate that commonly used instruments may not fully capture the psychological and situational demands associated with different sports disciplines and often lack athlete-relevant normative data, which can limit interpretability in applied settings [15,19]. In this context, the applicability of personality inventories depends on their empirical validation within specific cultural and athletic populations.

The NEO Personality Inventory and its revised version (NEO-PI-R) are among the most frequently used personality assessment tools in sport [20]. Although these instruments assess five broad personality traits, their administration can require up to 60 minutes, limiting their practicality in applied sport settings. Shorter instruments, such as the Mini-IPIP [21], offer greater efficiency but may provide less detailed assessment of personality constructs. Other measures, such as the Sports Mental Toughness Questionnaire (SMTQ), focus on more narrowly defined psychological characteristics rather than broad personality domains [22].

Many existing inventories were developed in specific cultural contexts and therefore require empirical evaluation when applied across different linguistic or national populations. This issue is particularly relevant in countries such as Latvia, where adaptations of instruments such as the NEO-PI-R and the Big Five Inventory have demonstrated limitations in cultural sensitivity and interpretive depth [23,24]. Taken together, these considerations highlight the need to examine how established personality instruments function within athletic and cultural contexts, providing the basis for evaluating and refining tools such as the Latvian Personality Inventory (LPI-v3) for use in sport-related research and practice.

### 1.3. The Latvian Personality Inventory (LPI-v3)

The LPI-v3 is a multidimensional personality assessment tool developed to measure a broad range of traits within the Latvian cultural context. It was created to address limitations in adapted instruments like the NEO-PI-R [22] and the Big Five Inventory [24], which showed reduced cultural sensitivity in applied settings. Specifically, in the Latvian version of the NEO-PI-R, several sub-scales did not significantly contribute to their theoretically intended factors [22].

To identify a personality structure suitable for the Latvian sociocultural environment, the development of the LPI-v3 followed a combined deductive–inductive and emic–etic approach, integrating theoretical elements from both the Five-Factor Model [20] and the HEXACO model [25]. The initial stage involved the generation of an extensive item pool based on 43 theoretically defined facets derived from both taxonomies. Working definitions were informed by psychological literature and the Latvian Language Dictionary [26]. To enhance clarity and applicability, items were formulated using simple language, avoiding complex or foreign terms and minimizing susceptibility to socially desirable responding [27].

The initial pool of 650 items underwent rigorous evaluation: ecological validity was assessed through six focus groups (one per domain) to ensure subjective comprehension, while content validity was evaluated by independent experts. This resulted in 398 items for empirical assessment [28]. Following two empirical studies (N_1_ = 820, N_2_ = 1294), the final 100-item version of the LPI-v3 was established. It consists of 24 facet-level scales (4 items each) grouped into six domains: (1) Neuroticism: N1: Anxiety, N2: Vulnerability, N3: Depressivity, N4: Diffidence; (2) Conscientiousness: C1: Orderliness, C2: Self-discipline, C3: Perfectionism, C4: Prudence; (3) Extraversion: E1: Sociability, E2: Joyfulness, E3: Sensation-seeking; E4: Social Boldness; (4) Agreeableness: A1: Flexibility, A2: Gentleness, A3: Obedience, A4: Composure; (5) Openness: O1: Aesthetic, O2: Social Tolerance, O3: Inquisitiveness, O4: Creativity; (6) Honesty-Humility: H1: Sincerity, H2: Greed-avoidance, H3: Modesty, H4: Fairness.

The LPI-v3 structure integrates elements from both major models: Neuroticism, Extraversion, and Conscientiousness largely match the FFM/NEO-PI-R, while Honesty-Humility aligns perfectly with the HEXACO model. In the Openness and Agreeableness domains, 75% of the facets correspond to HEXACO, with “Social Tolerance” replacing “Unconventionality” and “Obedience” replacing “Forgiveness” to better reflect local cultural nuances. Results of target rotation confirmed that the LPI-v3 inner structure is stable and replicable across different samples (stratified, male, and female), with insignificant differences in factor matrices [28].

While the inventory has demonstrated an interpretable factor structure and satisfactory internal consistency within the general population, its functioning has not yet been systematically examined in athletic populations. Given the specific psychological and contextual characteristics associated with sports participation, further psychometric evaluation is required to determine the suitability of the LPI-v3 for use among athletes and to support the development of population-specific normative data.

### 1.4. The present research: Study 1 and Study 2

Despite the availability of several personality inventories used in sport psychology, there remains a lack of culturally adapted and psychometrically validated personality instruments specifically evaluated in athlete populations within smaller linguistic contexts such as Latvia. In particular, limited evidence regarding measurement invariance and athlete-specific normative data restricts the interpretability and comparability of personality assessments in sport settings. The present research was designed to address this gap through the systematic validation and standardization of a context-sensitive personality measure developed for the target population. The present research comprised two sequential studies designed to empirically examine, refine, and apply a shortened version of the Latvian Personality Inventory (LPI-v3s) in an athlete population.

Study 1 focused on the psychometric evaluation and standardization of the instrument, with the aim of deriving a time-efficient measure and examining its factorial structure and measurement invariance in athletes. Study 2 built on these findings by exploring the criterion-related validity of the LPI-v3s, examining associations between personality traits and sport achievement level. Accordingly, the overarching aim of the present research was to evaluate the suitability of the LPI-v3s for use in athletic contexts by (a) examining its measurement structure and gender invariance (Study 1), (b) developing age- and gender-appropriate normative data where required (Study 1), and (c) exploring the extent to which specific personality traits are associated with elite/pre-elite versus non-elite sport achievement status (Study 2).

The main objectives of the study were as follows:

1. To derive a shortened version of the Latvian Personality Inventory (LPI-v3s) for use in athlete samples and examine its factorial structure and measurement invariance across male and female athletes (Study 1).
2. To determine which personality scales, require gender- and/or age-specific normative interpretation and to establish corresponding normative indicators (Study 1).
3. To explore associations between personality traits (at both trait and factor levels) and sport achievement status (elite/pre-elite vs. non-elite) (Study 2).

Despite the availability of a variety of personality inventories based on the Five-Factor Model, many have not been systematically evaluated in athlete populations and may show limited cultural sensitivity when applied in smaller language contexts such as Latvian. In addition, the factorial structure, measurement invariance, and normative applicability of these instruments are often assumed rather than empirically tested in sport samples. This highlights the need for psychometrically sound, time-efficient personality measures whose functioning is explicitly examined within athletic and cultural contexts relevant to sport psychology practice.

## 2. Methods and materials

### 2.1. Participants

The Initial Sample included 931 participants aged 15–45. Five participants who did not identify as male or female were excluded solely for methodological reasons, as gender was a primary stratification element for subsequent analyses. The remaining set of participants formed the total sample (*N* = 925). This sample was used as the source pool for: (1) selecting participants for the normative sample (*n* = 436) used in the development and psychometric validation of LPI-v3s (Study 1), and (2) forming the final analytical sample (*n* = 753) for the evaluation of the relationship between the athletes’ personality traits and sport achievement status (Study 2).

#### 2.1.1. Total sample (Study 1 and Study 2).

The total sample comprised participants, with ages ranging from 15 to 45 years (*M* = 21.2, *SD* = 5.6 years). The sample consisted of 535 males (57.8%), aged 15–45 years (*M* = 20.9, *SD* = 5.8), and 390 females (42.2%), aged 15–43 years (*M* = 21.1, *SD* = 4.8). Sports experience among the participants ranged from one year in specific sport to 35 years (*M* = 7.2 years, *SD* = 4.8 years), and training intensity ranged from 1 to 25 hours per week (*M* = 7.8 hours/week, *SD* = 4.8 hours/week); 490 (53.0%) participants represented Team sports (356 males, 134 females) and 435 (47.0%) participants represented Individual sports (179 males, 256 females). Sport Achievement Level initially was classified in three categories: elite (*n* = 46, 5.0%), pre-elite (*n* = 433, 46.8%), or non-elite (*n* = 446, 48.2%). Together, athletes represented 84 different sports, including basketball (n = 107), hockey (n = 96), football (n = 94), volleyball (n = 65), athletics (n = 56), fitness (n = 47), handball (n = 42), floorball (n = 41), luge sport (n = 31), swimming (n = 28), orienteering (n = 27), judo (n = 15), sports dances (n = 15), table tennis (n = 13), rugby (n = 13), weightlifting (n = 12), boxing (n = 10), artistic gymnastics (n = 10), cross-country skiing (n = 9), tennis (n = 9), karate (n = 9), cycling (n = 8), beach volleyball (n = 8), equestrian sport (n = 8), alpine skiing (n = 7), gymnastics (n = 6), kayaking (n = 6), taekwondo (n = 6), figure skating (n = 6), climbing sport (n = 5), running (n = 5), shooting (n = 5), biathlon (n = 4), bodybuilding (n = 4), curling (n = 4), triathlon (n = 4), road cycling (n = 4), disc golf (n = 4) and, other (n = 82).

Athletes’ competitive status was categorized based on training intensity and performance criteria. Elite athletes were defined as those who trained at least 8 sessions per week (or > 12 hours weekly) and had achieved at least one high-level competitive result, such as a podium at a national championship, a top placement in a regional league or participation in a major international competition (European Championships at junior or senior level, World Cup, World Championships, or Olympic Games). All athletes in the elite category also had a minimum of five years of sport-specific experience. pre-elite athletes trained at least 5 times per week (≈7.5 hours weekly) and had competitive experience at the national championship level (in any age category), in regional leagues, or at university-level events such as Universiade. Non-elite athletes engaged in a minimum of 2 training sessions per week (≈3 hours weekly), and their competition experience was limited to lower- or mid-tier competitions.

Missing data per participant did not exceed 5% and deletion was applied to maintain data integrity. Although the sample was broad and diverse, it represents a convenience sample of active athletes rather than a random selection from the national athlete population. The inclusion of participants aged 15 years and older reflects the study’s aim to examine personality measurement across key developmental stages in sport. Age-related differences were explicitly examined and addressed through stratified analyses and normative procedures rather than assumed equivalence across age groups.

#### 2.1.2. Normative sample (Study 1).

Participants from the total sample (*N* = 925) were stratified based on three criteria: gender (male/female), age (15–17, 18–20, 21–29, 30–45), and sport type (individual/team). The respondent distribution across these strata in the Analysis Sample was highly uneven. Since no official population data on the distribution of athletes by these specific characteristics was available, and because the primary aim of norm development was comparability rather than population representativeness, equal number of respondents per stratum were selected to ensure maximum structural uniformity and direct comparability across groups. This approach reflects a deliberate methodological trade-off between statistical balance and population representativeness. The equal-cell strategy was selected to ensure stable and comparable normative estimates across demographic strata, while recognizing that the resulting norms should be interpreted as balanced reference standards rather than direct representations of the natural distribution of athlete characteristics in the broader population.

Following the principle of the smallest available cell size, the final sample size for most strata was determined by the number of participants in the smallest available group, which was the female 15–17 age group in team sports (*n* = 32). To ensure a uniform representation across all other strata, a total of 32 participants were randomly selected from each of the remaining strata that had more than 32 available respondents. The randomization was performed using an online random number generator (e.g., randomizer.org), ensuring that each participant within a stratum had an equal chance of being selected for the final sample.

An exception was made for the 30–45 age group, which had a significantly smaller number of participants. To ensure sufficient power for the gender-specific norms, it was decided to use the highest number of available respondents for each gender within this age group. This meant including all 21 available male respondents in each of their respective sport type strata. For female respondents, due to the low number of participants in team sports (*n* = 5), a proportional number of participants (*n* = 5) were randomly selected from the female individual sports group to maintain proportional representation within the female 30–45 age group. This decision prioritized obtaining the largest possible sample size for each gender while preserving the sport type balance within the older female group. This approach prioritized the largest feasible sample size within older age strata while maintaining internal consistency of stratification criteria.

Therefore, to ensure minimally sufficient representation (or adequate sample sizes) within age strata for the development of both gender- and age-specific norms where required, a decision was made to consolidate the initial four age groups into two broader categories: adolescents/young adults (15–20 years) (combining 15–17 and 18–20 age groups) and Adults (21–45 years) (combining 21–29 and 30–45 age groups). This approach allowed for the standardization of LPI-v3s scores for males and females, with the flexibility to implement age-based norms for specific scales identified as being significantly influenced by age (as detailed in Section 2.2.3). The final composition of the normative sample is detailed in Appendix A Table S1 in S1 Appendix, which illustrates the initial and final counts for each stratum. The data from this normative sample (*n* = 436) were utilized to develop and offer a reliable measure of the abbreviated version of the LPI-v3s and establish the necessary normative scores for the athlete population (see Appendix A Table S2 in S1 Appendix).

#### 2.1.3. Final analytical sample and data quality control (Study 2).

The total sample (*N* = 925) was subsequently subjected to a final data quality control procedure to form the Final Analytical Sample used for the main predictive analyses. Crucially, scores on the Lie Scale (M) from the shortened version of the LPI-v3s (as detailed in Section 2.2.2.) were utilized as a criterion for data exclusion to ensure the validity of the personality profile analysis and minimize the impact of socially desirable responding within the final analytical sample. Participants who scored above the established cutoff on the Lie Scale (the T score > 60), commonly interpreted as indicating potentially distorted self-presentation, were identified as providing potentially invalid data. This threshold corresponds to scores exceeding one standard deviation above the normative mean and is widely used as a conservative criterion for data quality control. To evaluate the robustness of this decision rule, sensitivity checks were conducted using slightly alternative cutoff thresholds. These analyses indicated that the overall pattern of predictive results and model estimates remained substantively unchanged, suggesting that the exclusion criterion did not materially influence the main findings. Consequently, a total of 160 participants (≈17.3% of the sample) were excluded.

The final Analytical sample used for all subsequent statistical analyses (prediction of sport achievement) consisted of 753 participants (437 males and 316 females). Detailed distributions by sport type, performance level, and gender are presented in Appendix A Table S3 in S1 Appendix.

### 2.2. Measures

#### 2.2.1. The original version of the Latvian Personality Inventory (LPI-v3).

The Latvian Personality Inventory (LPI-v3) is a 100-item self-report questionnaire used to assess six personality factors: Neuroticism, Extraversion, Openness to Experience, Agreeableness, Conscientiousness, and Honesty-Humility [29]. The instrument is hierarchically structured, also measuring four narrower personality traits (facets) within each of the six factors. Each of the six factors is measured by 16 items, while each facet (subscale) is measured by 4 items. An additional 4 items form a Lie scale, which assesses the tendency toward socially desirable responding. Participants respond using a 5-point Likert scale ranging from 1 (“Does not correspond to me”) to 5 (“Corresponds to me”). Scale scores for both the personality factors and the facets were derived by summing the scores of the respective items, dividing the sum by the number of items (to yield the mean score), and finally multiplying this mean score by 10. The resulting scaled scores range from 10 to 50. Higher scores indicate a greater expression of the trait as defined by the scale name.

Previous research in general population samples has reported satisfactory internal consistency and an interpretable factor structure for the LPI-v3 in the general Latvian population sample. Internal consistency (Cronbach’s alpha, *N* = 1294) for the factor scales range from 0.81 to 0.88, and for the facet scales, from 0.61 to 0.86. The inventory also shows high test-retest reliability [29]. Retest coefficients (*n* = 166; mean interval of 19 weeks) ranges from 0.85 to 0.90 at the factor level and from 0.68 to 0.85 at the subscale level [28]. The complete LPI-v3 questionnaire (Latvian) and scoring key are provided in Appendix B.

#### 2.2.2. Shortened version of the LPI-v3 (LPI-v3s).

The data from the normative sample (*n* = 436) was utilized to develop an abbreviated version of the LPI-v3 empirically derived and evaluated for use in the athlete population represented in this study. The primary objective of developing this shortened version was to ensure it achieved measurement invariance across gender groups, which is a prerequisite for correctly comparing mean scores between male and female athletes. Furthermore, the short version was designed to be robust enough to allow for the development of gender-specific and, where necessary, age-specific norms tailored to this athletic population (Study 1). The subsequent analyses for predicting sports achievement status were based on the T-scores derived from the empirically evaluated short version of LPI-v3s (Study 2).

To ensure accurate interpretation of personality traits, the necessity for developing separate age-based norms (adolescents/young adults [14–19] vs. adults [21–45]) was examined. This decision was informed by a preliminary analysis of the normative sample (*n* = 436), with the goal of detecting systematic variation of traits across the lifespan. The analysis was conducted separately for males and females.

**Fig 1:**
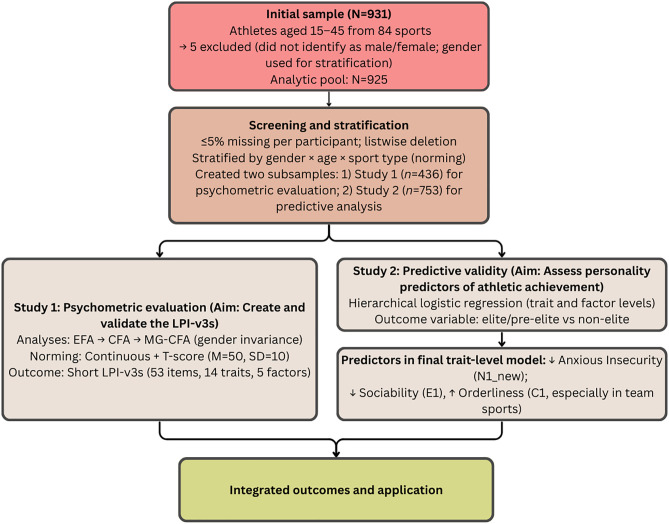
Overview of the research process (independent sample flow, psychometric evaluation, and predictive validity analysis).

A scale was deemed to require age-specific norms if: (1) Age correlated statistically significantly with the scale score (*p* < 0.05); and (2) The Independent Samples t-test or Mann-Whitney U test revealed a statistically significant mean difference (*p* < 0.05) between the 15–20 age group and the 21–45 age group, with a corresponding effect size of at least a small to moderate magnitude (e.g., Cohen’s *d* or *r* ≥ 0.20). Scales meeting both criteria were subsequently normed separately by age group, resulting in four distinct normative tables: (1) male adolescents/young adults, (2) male adults, (3) female adolescents/young adults, and (4) female adults. Fig 1 provides a visual summary of the sequential steps and methodological structure for the two-study research design, including sample flow, psychometric analyses, and predictive modeling. Raw-to-T conversions and continuous norm tables are available in Appendix C (Excel).

### 2.3. Study protocol and ethics

The data collection process for the LPI-v3 was conducted using a mixed-mode approach to ensure maximum reach and engagement among participants. Athletes were given the option to complete either a digitally accessible questionnaire through Microsoft Forms or a paper-based version, which was administered directly by members of the research team. The target population included athletes from various sports and competitive levels. Participant recruitment and data collection occurred over a 13-month period, beginning on 1 June 2024 and concluding on 1 July 2025. The inventory was distributed directly to athletes by the research team. In addition to completing the LPI-v3 and rating each item, participants were requested to share demographic details, such as their age, gender, city of residence, type of sport, hours spent training each week, highest achievements, and years of practicing in sport.

Participation in the study was anonymous and voluntary. All participants were informed that their data would be used exclusively for research purposes. Written informed consent was obtained prior to participation, ensuring that all individuals were aware of the study’s aims and how their data would be handled. In the case of participants under the age of 16, written informed consent was additionally obtained from their parents or legal guardians. The study was approved by the Ethics Committee of the Latvian Academy of Sport Education (Protocol No. 8, Statement No. 1, April 19, 2024) and was conducted in accordance with the ethical standards set forth in the Declaration of Helsinki.

Confidentiality was strictly maintained. All data were anonymized, encrypted, and stored on secure servers, accessible only to authorized members of the research team. The study adhered to applicable data protection regulations, with a registered data management plan submitted via the ARGOS (OpenAIRE) system. Participants were also informed of their right to withdraw from study at any time without penalty. In such cases, any identifiable data were immediately deleted to protect participant privacy.

### 2.4. Statistical analysis

Statistical analyses were performed separately for Study 1 (psychometric evaluation and norm development) and Study 2 (predictive validity). All analyses were conducted using JASP v0.95.4 and JAMOVI v2.6. Statistical significance was evaluated at p < 0.05, and effect sizes were reported where appropriate. Sample-size adequacy was verified using G*Power 3.1.9.6. A conservative sensitivity analysis assuming a statistical power of 90% (β = 0.10) and a significance level of α = 0.001 indicated that the final sample exceeded the minimum required size (*n* = 102) for adequate statistical sensitivity. Internal consistency was assessed using Cronbach’s α and McDonald’s ω coefficients, with values ≥ 0.70 considered acceptable.

#### 2.4.1. Study 1: Psychometric evaluation and norm development.

**Fig 2:**
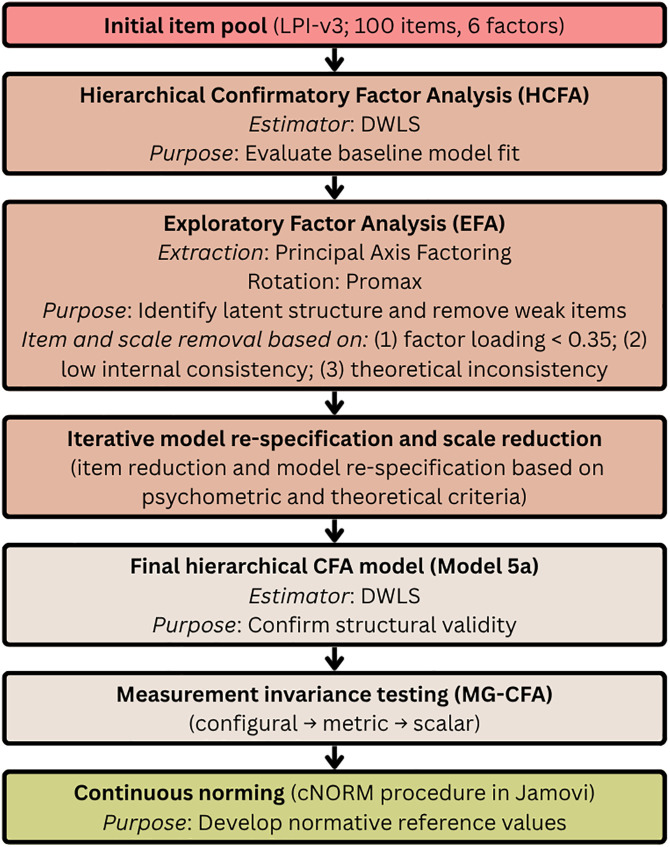
Analytical decision flow for factorial structure evaluation and model refinement in Study 1.

Factorial structure was evaluated iteratively. Initially, the model fit was tested on the full normative sample (*n* = 436) using hierarchical confirmatory factor analysis (HCFA). This was based on a polychoric correlation matrix and the diagonally weighted least squares (DWLS) estimator, which is robust for ordinal data. To identify a parsimonious structure, exploratory factor analysis (EFA) was subsequently conducted, employing principal axis factoring with Promax rotation at the item level and Varimax rotation at the scale level. The final configuration was then validated using the same HCFA procedure. To reduce the risk of sample-dependent optimization, item removal and model re-specification were guided by a combination of statistical criteria and theoretical interpretability, rather than solely by modification indices. All model refinement decisions were evaluated for conceptual consistency with the underlying personality framework, and the final structure was retained only when improvements in model fit were accompanied by stable factor interpretation and internal consistency. The sequential analytical steps and decision logic underlying the factorial structure evaluation and model refinement process are summarized in Fig. 2.

Model fit was evaluated using the Comparative Fit Index (CFI), Tucker–Lewis Index (TLI), Root Mean Square Error of Approximation (RMSEA, 90% CI), and Standardized Root Mean Square Residual (SRMR). Thresholds for acceptable fit were CFI and TLI ≥ 0.90, RMSEA ≤0.08, and SRMR ≤0.08. Given the complexity of hierarchical personality models and the number of observed indicators, slightly lower CFI/TLI values were interpreted cautiously and in conjunction with other fit indices and substantive interpretability, consistent with recommendations by previous research [30].

Measurement invariance across gender groups (male vs. female athletes) was examined using multigroup CFA. Configural, metric, and scalar invariance were tested sequentially. Invariance was supported when ΔCFI < 0.01 and ΔRMSEA < 0.015.

To determine the need for age-specific norms, Pearson’s correlations were calculated between age and personality-scale scores separately for males and females. Scales showing significant correlations (*p* < 0.05) were further compared between age groups (15–20 vs. 21–45 years) using independent-samples t-tests or Mann–Whitney U tests. Effect sizes were expressed as Cohen’s d (parametric) and rank-biserial *r* (non-parametric), with values ≥ 0.20 interpreted as practically meaningful.

Continuous norming was performed using the cNORMj package in Jamovi [31], supplemented by conventional T-score transformations (*M* = 50, *SD* = 10) to facilitate comparability across methods. The quality of norm models was verified by coefficients of determination (R² ≥ 0.996), indicating empirical equivalence between continuous and conventional norms. To minimize the risk of overfitting during model refinement, item removal decisions were guided by predefined statistical thresholds (factor loading < 0.35, internal consistency) and theoretical interpretability rather than post-hoc model fit optimization. Measurement invariance testing across athlete groups further supported the structural stability of the model. However, because no independent validation sample or split-sample cross-validation was available, the final measurement structure should be interpreted as provisional rather than definitively confirmed. The use of the same stratified normative sample for exploratory model refinement and subsequent confirmatory testing may have introduced some degree of sample-specific optimization; therefore, replication in independent athlete samples is required before stronger claims about the generalizability of the factorial structure can be made.

#### 2.4.2. Study 2: Predictive validity (trait and factorial level analysis).

Predictive validity of the LPI-v3s was evaluated using hierarchical binary logistic regression. Analyses were performed at two hierarchical levels: (1) Trait-level model: Step 1 included all trait-scale T-scores and sport type (Team = reference). Step 2 added interaction terms (Sport Type × Personality Trait) to test for moderating effects; (2) Factor-level model: A parallel analysis was conducted at the broader personality factor level.

Model performance was evaluated via Nagelkerke R^2^, overall classification accuracy, and Receiver Operating Characteristic (ROC) curves. Discrimination was interpreted using the Area Under the Curve (AUC), with thresholds of 0.60 = fair, 0.70 = good, ≥ 0.80 = very good. Sensitivity and specificity were calculated using a 0.50 cut-off. All predictive analyses were interpreted as exploratory and descriptive, given the measurement-development focus of Study 1.

## 3. Results

### 3.1. Study 1: Psychometric evaluation and norm Development of the LPI-v3s

#### 3.1.1. Initial model fit analysis.

Considering that the LPI-v3 was developed and standardized on a general population sample aged 18 and older, and this study’s data were collected from an athlete sample aged 15 and older, it was first necessary to confirm that the inventory’s theoretical model was appropriate for this dataset before calculating standardized scores. The model fit was initially tested on the full normative sample (*n* = 436) using hierarchical confirmatory factor analysis (HCFA).

The initial hierarchical model showed a poor fit to the data. The chi-square statistic was significant, χ² (4424) = 7484, p < .001, which is common in large samples. The Comparative Fit Index (CFI = 0.728) and Tucker–Lewis Index (TLI = 0.719) were below the acceptable threshold (> .90), indicating insufficient model fit. The Standardized Root Mean Square Residual (SRMR = 0.087) also exceeded the recommended limit (< 0.08), whereas the Root Mean Square Error of Approximation (RMSEA = 0.040; 90% CI [0.038, 0.041]) met the acceptable standard, suggesting a reasonable approximation of the population covariance matrix. Taking together, these indices indicated that the initial measurement model did not adequately represent the relationships among the observed variables in the normative sample. Therefore, further modifications were required to improve overall fit.

#### 3.1.2. Development and evaluation of modified models (Models 1–4).

To improve model fit, several modified models were sequentially tested, aiming to enhance the fit indices while retaining the original theoretical model as much as possible. This sequential process was guided primarily by the analysis of standard estimates of factor loadings and finally by modification indices.

In the first modified model, the O2 (Tolerance) scale was excluded from O (Openness) factor, as its standard estimate on the O factor was 0.153, which is too low. Additionally, eight other items were removed (J41 from the E2 (Joyfulness) scale, J3 from the H1 (Sincerity) scale, J59 from the H3 (Modesty) scale, J78 and J91 from the H4 (Fairness) scale, J64 from the N3 (Depressivity) scale and J37 because their standard estimates representing factor loadings were below 0.35. The fit indices improved slightly but as seen in Appendix A Table S4 in S1 Appendix, all indices except for RMSEA remained inadequate.

In the next step, using the same procedure, one more item was identified and removed: J15 from the H1 (Sincerity) scale. The fit indices for this second modified model also improved slightly but were still inadequate. Therefore, the model modification process continued.

Analyzing the second model, all items showed appropriate factor loadings, but four scales (A1: Flexibility, H3: Modesty, H4: Fairness and O2: Tolerance) showed unsatisfactory internal consistency metrics (Coefficient α and ω in the range of 0.381 to 0.591), indicating low reliability. Consequently, it was decided to exclude these scales from further analysis completely. This resulted in Modified Model 3. Although all fit indices for this model improved slightly (see Appendix A Table S4 in S1 Appendix), they still indicated inadequate model fit, with the exception of the RMSEA.

Analysis of the third modified model’s results showed that the H2 (Greed-Avoidance) scale’s factor loading in the H factor was inappropriate (λ = 0.287), leading to its removal from the H factor. However, since only one scale was left in this factor, it was also removed from the higher-order factors. But two separate scales (H1 and H2) were left in the model, which did not fit into any of the higher-order factors. Additionally, it was found that the J34 item had a reduced factor loading (λ = 0.366), and it was also excluded. Furthermore, at this stage, the proposed modification indices were carefully analyzed, and the model was supplemented with several relaxations, allowing four subscales to correlate with the N factor, one with the A factor, three with the C factor, and four with the O factor. Four correlations between the subscales were also permitted. All permitted correlations are theoretically justified and reflect complex relationships between personality factors and their constituent traits. As a result, the fourth modified model was obtained.

Despite slight improvements, this fourth modified model still revealed an unsatisfactory fit for the proposed model (CFI = 0.830) on the overall normative sample. This inadequate model fit was more pronounced in the male athlete subsample, where the CFI was below the 0.80 threshold, but a little better in the female subsample (CFI = 0.832) (see Appendix A Table S4 in S1 Appendix). A more detailed analysis revealed that in the male subsample, three items (J100 from O4: Creativity, J52 from N3: Depressivity, and J28 from H2: Greed-Avoidance) showed excessively low factor loadings, and some scales dropped from their factors (the E3 scale and O1: Aesthetic Interests scale). However, subsequent modifications to the model, including those based on modification indices, did not lead to significant improvements. The results remained illogical, at least with respect to some items and scales in the male subsample. Models 1–4 were tested sequentially to improve fit while retaining the original structure as far as possible. Respecifications were guided by standardized loadings (removing indicators < 0.35), internal consistency of facet scales, and only then modification indices. Across Models 1–4, fit improved but remained suboptimal, particularly in the male subsample (Table S4 in S1 Appendix). Therefore, we proceeded with an EFA-guided re-specification.

#### 3.1.3. EFA-guided model and final HCFA (Model 5a).

To better understand the underlying factor structure of the LPI-v3s and address the poor fit of the initial *a priori* models, an exploratory factor analysis (EFA) was performed on the overall normative sample (*N* = 436). The EFA utilized a polychoric correlation matrix and the Principal Axis Factoring method. The number of factors was determined using Kaiser’s criterion (eigenvalues > 1), which yielded a 14-factor solution for the personality traits, plus an additional 15th factor representing the Social Desirability (Lie – coded as M scale) scale (see Appendix A Table S5 in S1 Appendix). Promax rotation was applied to allow for correlations between factors, as facet-level personality scales are theoretically expected to overlap.

The EFA results revealed a more parsimonious structure, explaining 53.7% of the total variance, yet demonstrating significant deviations from the original 24-facet model. Seven scales were entirely eliminated due to poor psychometric performance, theoretical inconsistency, or problematic mergers: H1 (Sincerity), H3 (Modesty), H4 (Fairness), A1 (Flexibility), E4 (Social Boldness), O2 (Social Tolerance), and C3 (Perfectionism). Specifically, E4 was removed due to cross-loadings with O4 (Creativity), while C3 merged unacceptably with O3 (Inquisitiveness). In the Honesty-Humility domain, only the H2 (Greed-Avoidance) scale remained viable.

A significant structural shift occurred within the Neuroticism domain. While the original LPI-v3 designates four distinct facets (Anxiety, Vulnerability, Depression, and Diffidence), the EFA indicated that these traits merged into a single, unified factor—the Anxious-Insecurity scale (N1_new). This newly formed composite scale represents the broader Neuroticism domain and consists of nine items: all four items from the original Vulnerability (N2) facet, two items from Depression (N3), two items from Diffidence (N4), and one item from Anxiety (N1).

Regarding the final scale lengths, the Anxious-Insecurity scale consists of nine items, while five other personality facets and the Lie (M) scale retained all four of their original items. The remaining eight facets were reduced to three items each.

The internal consistency, measured by Cronbach’s alpha, was evaluated in light of these reductions. While scales with only three items exhibited slightly lower alpha coefficients, this decrease is mathematically consistent with the reduced item count, as internal consistency is inherently sensitive to scale length. Given the multidimensional nature of the LPI-v3s and its intended use for group-level comparisons and norming in athletic populations, these reliability levels were considered acceptable. This refined 15-factor measurement model served as the basis for the final Model 5a, subsequently subjected to confirmatory factor analysis and measurement invariance testing.

#### 3.1.4. Second-order factor analysis and domain structure.

To explore the higher-order structure of the LPI-v3s and determine how the identified facets group into broader personality domains, a second-order EFA was conducted. Given that the facet scores represent interval-level data, the analysis was based on a correlation matrix. The Principal Axis Factoring extraction method was employed, followed by a Varimax rotation to achieve a simple, independent factor structure. The optimal number of factors was determined using Parallel Analysis, which suggested a six-factor solution.

While the analysis generally supported the expected multi-factor structure, explaining 43.7% of the total variance, several deviations from the original taxonomies were observed (see Appendix A Table S6 in S1 Appendix). A notable finding concerned the E3 (Sensation-seeking) scale, which failed to load significantly on the general Extraversion (E) factor, suggesting that for this specific athletic population, physical risk-taking and excitement-seeking may function independently from social extraversion. Furthermore, the Honesty-Humility domain was significantly weakened; as H2 (Greed-Avoidance) was the only remaining facet of this factor, it lacked the necessary saturation to form an independent, stable H-domain. Consequently, the sixth factor identified by the Parallel Analysis was deemed uninterpretable and psychometrically unstable, leading to its exclusion from the final domain-level model.

The analysis also highlighted the complex nature of the A4 (Composure) scale. In line with the Five-Factor Model (FFM), this scale demonstrated a higher factor loading on the Neuroticism (N) factor when reversed, alongside the newly unified Anxious-Insecurity scale (N1_new). However, it also maintained a substantial loading on the Agreeableness (A) factor, as originally intended in the HEXACO framework. To preserve the psychological richness of this trait and ensure internal consistency within both domains, a dual-loading scoring decision was implemented. Specifically, the scale is scored in its reversed form as a new Neuroticism facet, N2_new (Irritability), reflecting emotional volatility, while concurrently being scored in its original form within the Agreeableness domain to reflect interpersonal composure. This refined 15-factor measurement model (including the Lie scale) served as the basis for the final Model 5a, which was subsequently subjected to confirmatory factor analysis and measurement invariance testing.

#### 3.1.5. Confirmatory factor analysis and model fit of the refined LPI-v3s.

Based on the EFA findings, a revised, shortened model was developed, comprising 53 items, 14 facets, and 5 higher-order factors and additional 4 items of validity scale (Lie scale) (not included in the CFA). This model was subsequently subjected to a hierarchical confirmatory factor analysis (CFA), designated as Model 5. To establish a baseline, the fit indices for this initial structural model were gathered. Following an evaluation of the modification indices and theoretical justifications, several correlations between specific scale residuals and factors were permitted (e.g., based on covariances > 0.35). This refined version, referred to as Model 5a, demonstrated a significantly improved fit to the data.

Although the CFI for Model 5a was 0.885 (see Table 1), it remained below the conventional threshold of 0.90. However, as Model 5a is significantly more parsimonious than the original version, it still retains considerable structural complexity. As noted by Marsh et al. [32,33] and McNeish and Wolf [34], psychometric instruments with a large number of scales and items often face difficulty in achieving the stringent benchmarks (CFI and TLI > 0.95) proposed by Hu and Bentler [30]. These authors argue that for complex personality constructs, fit indices around 0.80 or 0.85 can be considered acceptable. Given that the model was nearing the 0.90 threshold and provided a theoretically sound representation of the data, it was deemed suitable for further analysis.

**Table 1:** Goodness-of-fit indices of the HCFA modified Model 5a.

Model	Sample	*N*	*χ*^*2*^ (df)	p	CFI	TLI	RMSEA (90% CI)	SRMR
*Modified Model 5*	Normative sample	436	2505 (1291)	< 0.001	0.853	0.843	0.046 [0.044; 0.049]	0.074
*Modified Model 5a* ***(final retained model)***	Normative sample	436	2217 (1276)	< 0.001	0.885	0.876	0.041 [0.038; 0.044]	0.068
*Modified Model 5a*	Male athletes	234	1857 (1276)	< 0.001	0.849	0.837	0.044 [0.040; 0.049]	0.085
*Modified Model 5a*	Female athletes	202	1662 (1276)	< 0.001	0.878	0.868	0.039 [0.033; 0.044]	0.083

*Note*. Estimator is DWLS. Model test is scaled and shifted. Information matrix is expected. Standard errors are robust. Fit indices are based on the scaled test statistics. χ²/df: Degrees of freedom; CFI: Confirmatory Fit Index; TLI: Tucker-Lewis Index; RMSEA: Root Mean Square; SRMR: Standardized Root Mean Square Residual. The final retained model is highlighted in bold to facilitate interpretation of the primary analytical results.

The primary objective of this CFA was to identify a measurement model suitable for measurement invariance testing a critical prerequisite for valid gender-based norming and comparisons of personality traits in athletic performance. Model 5a’s fit indices for the overall normative sample and the separate male and female subsamples are presented in Table 1. The indices were higher in the female subsample compared to the male subsample (CFI = 0.878 and 0.849, respectively), though both were lower than in the combined normative sample. Nevertheless, these values represented a significant improvement over previous iterations and satisfied the minimum criterion of > 0.80, which is acceptable for such complex survey structures.

The decision to utilize the same stratified normative sample for both the EFA and the subsequent CFA was driven by the need to maintain the psychometric integrity of the gender- and age-balanced group required for norming. The full stratified sample provided the necessary statistical power to identify a stable structure and immediately verify its suitability for invariance testing. This approach ensured that the resulting measurement model provided a robust foundation for the planned gender-based comparisons. Fit indices for the initial refined model (Model 5), the final retained model (Model 5a), and its performance across the overall, male, and female subsamples are presented in Table 1. For readability, the key result in Table 1 is the final retained Model 5a in the total normative sample, which showed improved but still moderate fit (CFI = 0.885, TLI = 0.876, RMSEA = 0.041, SRMR = 0.068). The male and female subgroup results are presented to demonstrate the stability of the retained model across gender groups, rather than as separate alternative models.

Due to its enhanced parsimony and improved, although not optimal, fit indices, Model 5a was adopted as a provisional measurement model for the LPI-v3s. This structure was deemed sufficiently robust to serve as the basis for subsequent measurement invariance testing and the development of normative data (Study 1), as well as for the predictive validity analyses conducted in Study 2.

#### 3.1.6. Measurement invariance.

Factorial invariance was tested using multigroup confirmatory factor analysis (MG-CFA) to determine if the measurement model for the modified shortened version (Model 5a) of LPI-v3s was equivalent across male and female athlete samples. The analysis followed a hierarchical approach, starting with the least restrictive model and progressing to more restrictive models. The goodness-of-fit was evaluated using the chi-square statistic (χ^2^), degrees of freedom (df), comparative fit index (CFI), Tucker-Lewis Index (TLI), and the root mean square error of approximation (RMSEA) with its 95% confidence interval. A significant drop in fit for the more restricted models was assessed using the change in CFI (ΔCFI), with a value less than 0.01 considered acceptable [35,36].

The first step was to test for configural invariance, which assesses whether the factor structure is the same across both groups without imposing equality constraints on the model parameters. The results of the configural model indicated an acceptable, but not excellent, fit to the data (χ^2^(2552) = 3516; p < 0.001). The fit indices were CFI = 0.863, TLI = 0.852, and RMSEA = 0.042 [0.038; 0.045]. These results support the notion that the same number of factors and the same pattern of factor loadings were present in both male and female samples.

Following the establishment of configural invariance, metric invariance was tested by constraining the factor loadings to be equal across both groups. This model showed a slight deterioration in fit compared to the configural model (χ^2^(2603) = 3553; *p* < 0.001). The change in fit was minimal (Δχ^2^(51) = 37; ΔCFI = 0.002), which is below the recommended threshold of 0.01. This suggests that the factor loadings are equivalent across both male and female samples, supporting the comparability of the factor-item relationships between the two groups.

The final step was to test for scalar invariance by adding the constraint that the item interceptions are equal across both groups. This model provides the strongest test of measurement equivalence. The results showed a significant drop in model fit compared to the metric invariance model based on the chi square test (χ^2^(2741) = 3746; *p* < 0.001), nevertheless, the change in CFI was 0.008 (ΔCFI = 0.008), which is below the recommended threshold of 0.01. This suggests that the item interceptions are equivalent, allowing for direct comparison of latent mean scores between the groups. Based on the ΔCFI criterion, scalar invariance was supported (see Table 2), indicating that gender-based score comparisons are permissible within the present sample. However, this result should be interpreted in the context of the provisional measurement model and requires replication in independent athlete samples.

**Table 2:** Sequential tests of measurement invariance across gender groups for the LPI-v3s.

Model	χ2	df	CFI	TLI	RMSEA (95% CI)	Δ χ2 (Δdf)	ΔCFI
*Configural invariance*	3516	2552	0.863	0.852	0.042 [0.038; 0.045]	--	--
*Metric invariance*	3553	2603	0.865	0.857	0.041 [0.038; 0.044]	37 (51)	0.002
*Scalar invariance* ***(final model)***	3746	2741	0.857	0.85	0.04 [0.038; 0.044]	193 (138)	0.008

*Note*. χ²: Chi-square; df: degrees of freedom; CFI: Comparative Fit Index; TLI: Tucker–Lewis Index; RMSEA: Root Mean Square Error of Approximation; Δχ² = Chi-square difference test; Δdf = difference in degrees of freedom; ΔCFI = change in Comparative Fit Index. The primary criterion for evaluating invariance was ΔCFI < 0.01. The key finding is that the scalar model remained within this threshold (ΔCFI = 0.008), supporting gender-based comparisons within the present sample.

#### 3.1.7. Gender differences and decision for gender norms.

To determine which scales require gender-specific norms, mean differences between male and female athletes were examined using the Mann-Whitney U test due to non-normality across most scales.

The analysis identified statistically significant differences (*p* < 0.05) in the central tendency (median) for six scales. However, only five scales demonstrated a meaningful effect size (*r* > 0.20): N1_new (Anxious Insecurity), N2_new (Irritability), N (Neuroticism), A4 (Composure), and O1 (Aesthetic Interests). The largest effect sizes were observed for N (*r* = 0.32) and N1_new (*r* = 0.29).

Based on the combined criteria of statistical significance and meaningful effect size, gender-specific norms were deemed necessary for the five identified scales. All remaining scales (including those that reached statistical significance but had *r* < 0.20) showed negligible differences between male and female athletes, suggesting that unified norms can be applied for these traits (see Table 3).

**Table 3:** Mean differences and effect sizes between gender groups in the LPI-v3s normative sample.

LPI-v3 Scale (Short Form)	Mean (*SD*) normative sample (*n* = 436)	Mean (*SD*) male (*n* = 234)	Mean (*SD*) female (*n* = 202)	Test statistic (*U*)	*p*-value	Effect size *r*	Norms required
N1_new: Anxious-Insecurity	26.3 (7.3)	24.5 (6.6)	28.3 (7.4)	16719	< 0.001	0.29	Yes
N2_new: Irritability	27.9 (9.7)	26.0 (9.3)	30.0 (9.7)	18213	< 0.001	0.23	Yes
E1: Sociability	30.0 (9.1)	29.6 (9,0)	30.4 (9.3)	22899	0.573	0.03	No
E2: Joyfulness	37.8 (7.6)	38.1 (7.5)	37.6 (7.8)	22743	0.493	−0.04	No
E3: Sensation-Seeking	34.6 (8.5)	34.9 (8.1)	34.3 (8.9)	22930	0.590	−0.03	No
C1: Orderliness	33.9 (8.3)	33.8 (8.4)	34.3 (8.3)	23402	0.859	0.01	No
C2: Self-discipline	32.2 (8.5)	31.6 (4.9)	31.0 (9.2)	19941	0.005	−0.16	No
C4: Prudence	32.1 (8.0)	33.2 (7.6)	31.8 (7.6)	22487	0.378	−0.05	No
A2: Gentleness	32.8 (8.4)	32.6 (8.4)	33.0 (8.4)	23120	0.694	0.02	No
A3: Obedience	32.8 (5.7)	32.5 (6.1)	33.1 (5.1)	22668	0.453	0.04	No
A4: Composure	32.1 (9.7)	34.0 (9.3)	30.0 (9.7)	18213	< 0.001	−0.23	Yes
O1: Aesthetic Interests	24.9 (9.4)	23.2(8.7)	26.8 (9.8)	18533	< 0.001	0.22	Yes
O3: Inquisitiveness	32.2 (7.8)	32.6 (7.7)	31.7 (7.9)	22603	0.430	−0.04	No
O4: Creativity	32.5 (8.0)	32.8 (7.1)	32.2 (8.9)	22882	0.564	−0.03	No
H2: Greed-Avoidance	30.8 (8.7)	30.1 (9.0)	31.6 (8.3)	21168	0.058	0.10	No
N: Neuroticism	26.8 (6.8)	25.0 (6.3)	28.8 (6.8)	15957	< 0.001	0.32	Yes
E: Extraversion	33.9 (6.9)	33.8 (6.7)	34.0 (7.1)	23461	0.895	0.01	No
C: Conscientiousness	32.9 (6.4)	33.2 (6.5)	32.5(6.2)	21972	0.205	−0.07	No
A: Agreeableness	32.4 (6.5)	32.9 (6.4)	31.9(6.6)	21820	0.166	−0.08	No
O: Openness to Experience	30.1 (6.1)	29.8 (5.4)	30.4(6.7)	22394	0.344	0.05	No
*M: Lie Scale*	*25.1 (7.2)*	*25.0 (6.9)*	*25.1(7.5)*	*23470*	*0.900*	*0.01*	*No*

#### 3.1.8. Age effect and decision for age norms.

Correlations with age (Criterion 1). To empirically determine the necessity of establishing age-based norms, Pearson correlational analyses were conducted between respondent age and all shortened version of the LPI-v3s scales’ scores within the normative sample, with analyses performed separately for the female and male subsamples. The results are detailed in (see Appendix A Table S7 in S1 Appendix).

Findings in the female subsample. Age was statistically associated with two factor-level scales and four facet scales in the female subsample. Consistent with general population trends, a negative, weak but statistically significant correlation was between age and Neuroticism (*r* = −0.18, *p* = 0.012). This relationship was reflected in the facet Anxious-Insecurity (*r* = −0.18, *p* = 0.009), suggesting that women tend to report lower levels of emotional instability or worry with increasing age. Similarly, Sensation-Seeking showed a small, significant decline with age (*r* = −0.19, *p* = 0.006). Conversely, a positive, weak but statistically significant correlation was found between age and Conscientiousness (*r* = 0.20, *p* = 0.005), which was primarily driven by the facet Orderliness (*r* = 0.19, *p* = 0.008), and weakly by Prudence (*r* = 0.14, *p* = 0.046).

*Findings in the male subsample* The male subsample displayed more numerous and often stronger correlations between age and personality traits, suggesting a greater influence of age on self-reported scores in this group. Positive, statistically significant correlations were found with the following factors:

1. Openness to Experience (*r* = 0.23, *p* < 0.001), which was largely accounted for by the facet Inquisitiveness (O3) (*r* = 0.28, *p* < 0.001).
2. Conscientiousness (*r* = 0.15, *p* = 0.022), which was largely accounted for by the facet Prudence (C4) (*r* = 0.22, *p* < 0.001), indicating increased cautiousness in decision-making with age.
3. The facet Greed-Avoidance (*r* = 0.31, *p* < 0.001) under the Honesty-Humility factor, which was the strongest correlation observed across all scales, indicating that male athletes report becoming substantially less status-conscious and more modest as they age.

Only two negative correlations in the male group were observed for the Agreeableness factor (*r* = −0.17, *p* = 0.08), which was largely accounted for by the facet Obedience (A3) (*r* = −0.18, *p* < 0.001), suggesting that older male athletes may be less inclined to defer to others or compromise compared to their younger counterparts.

*Mean differences between age groups* Following the correlational analysis which served as the first filtering criterion for age-specific norms, the second criterion for requiring age-specific norms was tested. Independent Samples t-tests (or the Mann-Whitney U test, based on normality assumptions) were performed only on those scales that had shown a statistically significant correlation with age in the respective gender subsamples. This step was necessary to confirm that the observed relationship translated into a meaningful mean difference (criterion) between the newly defined age groups: adolescents/young adults (15–20) and adults (21–45).

Prior to conducting mean difference analyses, assumptions of normality (Shapiro-Wilk test) and homogeneity of variances (Levene’s test) were examined for all scales demonstrating a significant correlation with age in their respective gender subsamples.

In the female subsample, Levene’s test indicated that the assumption of homogeneity of variances was met for all scales (*p* > 0.05). However, the normality assumption was violated for the Sensation-Seeking (E3) and Orderliness (C1) scales (*p*_Shapiro_ < 0.05). Consequently, mean differences for these two non-normally distributed scales were analyzed using the non-parametric Mann-Whitney U test, while the remaining scales were analyzed using the Independent Samples t-test (assuming equal variances).

A similar pattern was observed in the male subsample: Levene’s test confirmed the homogeneity of variances assumption for all correlating scales (*p* > 0.05). However, the Shapiro-Wilk test revealed a violation of the normality assumption (*p*_Shapiro_ < 0.05) for four facets: Prudence (C4), Obedience (A3), Inquisitiveness (O3), and Greed-Avoidance (H2). Therefore, the Mann-Whitney U test was applied to these four non-normally distributed scales, and the remaining scales were tested using the Independent Samples t-test (assuming equal variances).

*Female subsamples mean differences*. For the female subsample, five scales met the initial correlation criterion (*p* < 0.05). Subsequent mean comparison tests (Independent Samples -test or Mann-Whitney test, based on normality) confirmed that four out of five scales met the dual criteria for requiring age-specific norms (i.e., < 0.05 AND *d* or *r* ≥ 0.20) (see Appendix A Table S8 in S1 Appendix):

1. Neuroticism (*t* (200) = 3.35, *p* < 0.001, *d* = 0.49) and its facet Anxious-Insecurity (N1_new) (*t* (200) = 2.79, *p* < 0.006, *d* = 0.41) showed higher scores among younger women, indicating greater emotional stability with age.
2. Conscientiousness (*t* (200) = −2.97, *p* < 0.001, *d* = −0.43) and its facet Orderliness (C1) (*U* = 3775, *p* = 0.013, *r* = −0.20) showed higher scores among adult women, suggesting increased organization and diligence with age.

The Sensation-Seeking facet (E3) failed to meet the effect size criterion (*r* = − 0.08), despite the statistically significant value (*p* = .033), and thus did not warrant age-specific norms.

*Male Subsample Mean Differences*. In the male subsample, seven scales met the initial correlation criterion. Mean comparison tests confirmed that six out of the seven scales met the criteria for requiring age-specific norms (see Appendix A Table S9 in S1 Appendix):

1. Conscientiousness (*t* (232) = −2.60, *p* = 0.01, *d* = −0.34) and its facet Prudence (C4) (*U* = 5167, *p* < 0.001, *r* = 0.24) showed higher scores among adult men, indicating increased responsibility and long-term planning with age.
2. Obedience (A3) (*U* = 4920, *p* < 0.001, *r* = −0.27) showed lower scores among adult men, confirming the finding from the correlation analysis that older male athletes report being less yielding.
3. Openness (*t* (232) = −2.14, *p* = 0.034, *d* = −0.28) and the facet Greed-Avoidance (H2) (*U* = 4348, *p* < 0.001, *r* = 0.32) also met the criteria.

The Inquisitiveness facet (O3) failed to meet the effect size criterion (*r* = 0.19) with the established cut-off of r > 0.20, despite the statistically significant p-value (*p* = 0.014). Similarly, the Agreeableness factor (A) failed to meet the statistical significance criterion (*p* = 0.103), despite meeting the effect size criterion (*d* = 0.21). Therefore, these two scales did not warrant age-specific norms.

Based on the sequential analyses, age-specific norms were established for four scales in the female subsample and six scales in the male subsample. For all other scales, a single, gender-specific norm was retained, as these traits did not show a simultaneous statistically significant and practically meaningful change across the two age groups.

#### 3.1.9. Development of norm construction.

Normative scores for the LPI-v3s scales were calculated using a dual approach to ensure both high-quality norm tables and simplified scoring for subsequent analysis.

For the creation of the final norm tables, a regression-based continuous norming approach was employed, utilizing the cNORMj for single group module within the JAMOVI statistical software. This method offers significant advantages over conventional approaches, as it models the relationship between raw scores and normative scores using polynomial regression, thereby smoothing the percentile curve and eliminating gaps or abrupt changes that often occur with traditional percentile calculations [37]. Although the continuous function for age-normed scores was constrained to predefined groups (due to the single-group approach), this method effectively optimized the endpoints and filled any sparsity in the data.

For the purposes of the subsequent analytical steps (e.g., regression analysis) involving the Total Analytic Sample, T-scores were calculated using the conventional approach (using a linear transformation where the mean is set to *M* = 50 and the standard deviation to *SD* = 10).

Based on the preliminary statistical analysis of the athlete sample, it was determined that gender- and/or age-specific norms were necessary only for a subset of the scales. Specifically, gender-specific norms were developed for the following scales: N1_new, N2_new, N, C1, C4, C, A3, A4, O1, O and H2. Furthermore, a detailed review of the data indicated the need for age-specific norms within genders for several key traits:

1. For female athletes, separate norms for two age groups were created for N1_new, N, and C1.
2. For male athletes, separate norms for two age groups were created for A3, O, and H2.

For the remaining scales (E, E1, E2, E3, C2, A2, O3, O4, A, and M), a unified set of norms was developed, without subdivision by age or gender, as no statistically significant differences were observed across these demographic variables.

The cNORMj package allows for a direct comparison between conventional norms and the regression-based continuous norms. The quality of the continuous norming model was excellent, with the final model’s being no lower than 0.996 for all individual norm tables. This high degree of fit indicates that the two methods are empirically equivalent, and that no systematic error exists between the linear transformation and the regression model. Therefore, the T-scores calculated using the conventional approach are considered equivalent to those that would be assigned based on the newly created continuous norm tables, justifying their use in the primary data analysis.

### 3.2. Study 2: Predictive validity of the LPI-v3s for sport achievement

A hierarchical binary logistic regression was performed to examine the unique contributions of personality traits, sport type, and their interaction on the prediction of elite/pre-elite status (see Appendix A or coding and full results). The elite and pre-elite groups were merged into a single elite/pre-elite achievement category due to the limited number of participants in the elite category (*n* = 34). Insufficient sample size in the highly specific elite group would have compromised the statistical power and stability of the regression model’s parameter estimates, particularly when analyzing complex interactions. Merging these two categories, which represent the highest levels of competitive performance, ensured adequate cell sizes for robust analysis while still maintaining a meaningful distinction from the non-elite group.

*Model 1: Personality Traits and Sport Type (Main Effects)* Step 1 included the main effects of all personality traits and the Sport Type variable. The initial model was found to be statistically significant, χ^2^ (15) = 26.3, *p* = 0.035, the model accounted for 4.58% of the variance in sport achievement (Nagelkerke *R*^2^ = 0.046). In this first step, the significant predictors were:

1. Anxious Insecurity (N1_new): Significantly reduced the odds of being in the higher achievement group (OR = 0.96, *p* = 0.004).
2. Sociability (E1): Significantly reduced the odds of being in the higher achievement group (OR = 0.98, *p* = 0.013).
3. The main effect of sport type was not significant (OR = 0.43, *p* = 0.577).

*Model 2: Interaction Effects* Step 2 introduced the interaction terms (sport type x personality traits) to the model from Step 1. The addition of the interaction terms resulted in a non-significant improvement in the model’s overall fit, Δχ^2^(14) = 20.8, *p* = 0.107. The full model was statistically significant (Model 2), χ^2^(29) = 47.1, *p* = 0.018, and accounted for a slightly larger proportion of the variance (Nagelkerke *R*^2^ = 0.081). After the inclusion of the interaction terms, the following results were observed:

1. Main Effects (Model 2). The significance and direction of the main effects of Anxious Insecurity (OR = 0.96, *p* < 0.001), Sociability (OR = 0.97, *p* = 0.004), remained consistent with Model 1, but this time Orderliness became a significant predictor (OR = 1.03, *p* = 0.018)
2. Interaction Effects: The only statistically significant interaction term was Sport Type x Orderliness (C1) (OR = 0.965, *p* = 0.023). The negative coefficient (B = −0.04) indicates that the positive relationship between Orderliness and elite/pre-elite status (as evidenced by the main effect *B* = 0.03) is significantly attenuated (reduced) for athletes in Individual Sports compared to those in Team Sports (the reference category). The effect of Orderliness becomes non-significant (or slightly negative, *B* = −0.01) within the Individual Sport group. The remaining interaction terms were not statistically significant (*p* > 0.05)

*Predictive performance of the Second Model* The full model correctly classified 61.1% of cases. The overall predictive power of the final model was assessed using the ROC curve, yielding an Area Under the Curve (AUC) of 0.642 which indicates acceptable (or fair) discrimination. The model achieved a Sensitivity (correctly identifying elite/pre-elite athletes) of 72.7% and a Specificity (correctly identifying non-elite athletes) of 47.6 (using a cut-off value of 0.5) (see Table 4).

**Table 4:** Summary of hierarchical binary logistic regression analysis predicting sport achievement level.

Variable	Estimate (B)	SE	*p*	Odds Ratio (OR)	95%CI for OR
Intercept	2.75	1.57	0.079	15.67	[0.73, 337.35]
N1: Anxious-Insecurity	−0.04	0.01	< 0.001	0.96	[0.94, 0.98]
N2: Irritability	0.01	0.01	0.251	1.01	[0.99, 1.03]
E1: Sociability	−0.03	0.01	0.004	0.97	[0.95, 0.99]
E2: Joyfulness	0.01	0.01	0.277	1.01	[0.99, 1.03]
E3: Sensation-Seeking	0.00	0.01	0.632	1.00	[0.98, 1.01]
C1: Orderliness	0.03	0.01	0.018	1.03	[1.01, 1.06]
C2: Self-discipline	0.01	0.01	0.397	1.01	[0.99, 1.03]
C4: Prudence	−0.01	0.01	0.102	0.99	[0.97, 1.00]
A2: Gentleness	0.00	0.01	0.887	1.00	[0.98, 1.02]
A3: Obedience	0.00	0.01	0.528	1.00	[0.99, 1.02]
O1: Aesthetic Interests	−0.01	0.01	0.301	0.99	[0.98, 1.01]
O3: Inquisitiveness	−0.01	0.01	0.284	0.99	[0.97, 1.01]
O4: Creativity	0.00	0.01	0.791	1.00	[0.99, 1.02]
H2: Greed-Avoidance	0.00	0.01	0.547	1.00	[0.98, 1.01]
Sport type: individual – team	−0.85	1.52	0.577	0.43	[0.02, 8.40]
N1 ✻ Sport Type	0.031	0.02	0.107	1.03	[1.00, 1.06]
N2 ✻ Sport Type	0.020	0.02	0.306	1.02	[0.98, 1.06]
E1 ✻ Sport Type	0.020	0.02	0.224	1.02	[1.00, 1.06]
E2 ✻ Sport Type	0.003	0.02	0.880	1.00	[0.97, 1.04]
E3 ✻ Sport Type	0.022	0.02	0.189	1.02	[0.99, 1.06]
C1 ✻ Sport Type	−0.038	0.02	0.034	0.96	[0.94, 1.00]
C2 ✻ Sport Type	0.033	0.02	0.085	1.03	[1.00, 1.07]
C4 ✻ Sport Type	−0.017	0.02	0.354	0.98	[0.95, 1.02]
A2 ✻ Sport Type	−0.014	0.02	0.445	0.99	[0.95, 1.02]
A3 ✻ Sport Type	0.001	0.01	0.945	1.00	[0.98, 1.03]
O1 ✻ Sport Type	−0.002	0.02	0.927	1.00	[0.97, 1.03]
O3 ✻ Sport Type	−0.007	0.02	0.701	0.99	[0.96, 1.03]
O4 ✻ Sport Type	0.013	0.02	0.473	1.01	[0.98, 1.05]
H2 ✻ Sport Type	−0.002	0.02	0.877	1.00	[0.97, 1.03]

*Note.* The table displays the results of the second model. Personality scales were measured using T-scores. Estimates represent the log odds of “performance level = 1  = elite/pre-elite” vs. “performance level  = 0  = non-elite (reference group). Sport type was coded 0  = team sport (reference group) and 1  = individual sport.”

A parallel hierarchical binary logistic regression was conducted at the broader personality factor level. The full factor-level model demonstrated significance, χ^2^(11) = 26.00, *p* = 0.006. However, only the Neuroticism factor emerged as a statistically significant predictor of elite/pre-elite status (OR = 0.97, *p* = 0.001), indicating that higher Neuroticism slightly reduced the odds of being in the higher achievement group. Crucially, none of the interaction terms between personality factors and sport type were found to be statistically significant (*p* > 0.05).

The predictive performance of this factor-level model was slightly weaker compared to the trait-level model. The model correctly classified 58.6% of cases. The overall predictive power was assessed by the AUC of 0.605, indicating acceptable discrimination. The model achieved a Sensitivity of 77.1% and a Specificity of 36.9% (using a cut-off value of 0.5).

## 4. Discussion

### 4.1. Psychometric evaluation and structural validity

The aim of this study was to empirically examine and refine the psychometric functioning of the LPI-v3 within an athlete population by deriving and standardizing a shortened version (LPI-v3s), and to explore group-level variation to support context-appropriate personality assessment in athletic settings. From both applied experience and previous research, it has been noted that lengthy psychometric instruments may compromise data quality due to respondent fatigue [38,39]. Accordingly, there has been an increasing emphasis on the development of abbreviated personality inventories that improve feasibility while retaining sufficient psychometric integrity. However, many short instruments, such as the MINI-IPIP, achieve efficiency at the expense of construct breadth and interpretive depth.

The present findings provide evidence supporting the factorial structure of the LPI-v3s in an athlete sample, with generally acceptable model fit indices and adequate internal consistency across scales. Although the overall pattern of fit indices supported the structural validity of the model, the CFI remained slightly below the conventional threshold of 0.90. Accordingly, the structural validity of the LPI-v3s should be interpreted as acceptable but not optimal, and the current model should be considered provisional pending further validation in independent athlete samples. This means that the present findings provide initial support for the measurement structure of the LPI-v3s in Latvian-speaking athletes, but they should not be interpreted as final confirmation of the factorial structure. Measurement invariance analyses further indicated that the factor structure and item functioning were comparable across male and female athletes, suggesting that observed score differences can be interpreted meaningfully across gender groups. Measurement invariance implies that constructs are measured in a comparable manner across groups, reducing the likelihood that group differences reflect measurement artifacts rather than substantive variation [40,41]. In line with broader literature demonstrating the cross-cultural robustness of the Five-Factor Model [42,43], these results suggest that the LPI-v3s offers a psychometrically coherent representation of personality traits within the specific context of athletic populations. Importantly, the observed group-level variation underscores the relevance of demographic differentiation when interpreting personality scores in sport.

### 4.2. Age and gender-related variations and norm development

Previous research has documented systematic age-related variation in several personality dimensions among athletes, suggesting that age should be considered when interpreting personality scores in sport contexts [44,45]. Consistent with this literature, the present findings indicated meaningful age-related differences on selected LPI-v3s scales, supporting the empirical examination of age-specific normative interpretation rather than the assumption of developmental equivalence across age groups.

In the current sample, younger athletes aged 15–20 years exhibited higher Neuroticism and lower Conscientiousness scores compared to athletes aged 21–45 years. Similar age-related patterns have been reported in both athletic and non-athletic populations and are commonly attributed to ongoing psychological development during adolescence [46,47]. Importantly, these findings should be interpreted descriptively, as the present study did not directly assess underlying regulatory mechanisms.

Gender-specific age trends were also observed. Among female athletes, Neuroticism particularly the Anxiety–Insecurity facet—tended to decrease with age, while Conscientiousness increased, indicating greater emotional stability and task-oriented behavior in older age groups. Among male athletes, age-related increases were observed for Openness to Experience, whereas Agreeableness showed a tendency to decline with age. These patterns are broadly consistent with previous findings suggesting that personality development in sport may follow partially gender-specific trajectories influenced by social, competitive, and contextual factors [48,49].

Taken together, these results highlight the importance of considering age and gender when interpreting personality scores in athletic populations. Rather than implying fixed developmental norms, the age- and gender-specific reference values derived in this study provide a contextual framework for interpreting personality variation within the sampled athlete population. The inclusion of adolescent athletes further underscores the need for cautious interpretation, as personality traits during this period remain dynamic and sensitive to environmental influences.

### 4.3. Structural modification and the absence of the Honesty–Humility domain

The refinement of the LPI-v3s was guided by the principle of parsimony, prioritizing structural stability and internal consistency over broad domain coverage. While several scales were eliminated, the resulting configuration demonstrates high empirical clarity, yielding a robust framework consisting of five broad domains and two distinct facet-level scales: Sensation-Seeking (E3) and Greed-Avoidance (H2).

The removal of scales such as Social Boldness (E4) and Perfectionism (C3) was necessitated by problematic cross-loadings that previously obscured the core dimensions. By refining the model, we achieved a structure where each item uniquely contributes to its respective factor (loadings > 0.45), thereby strengthening the theoretical interpretability of the remaining domains. The primary factor, Anxious-Insecurity (Factor 1), exemplifies this structural integrity, with nine items loading consistently above 0.55, prioritizing dimensions with the highest diagnostic value in an athletic population.

Beyond these primary shifts, the elimination of scales such as Sincerity (H1), Modesty (H3), Fairness (H4), Flexibility (A1), and Social Tolerance (O2) was driven by poor psychometric performance specifically within the athletic context. These scales exhibited high uniqueness values and low factor loadings, suggesting they did not coalesce into stable latent constructs in this sample. For instance, the lack of stability in Flexibility and Social Tolerance might reflect the highly structured and rule-bound nature of competitive sports, where these traits may be subject to different normative pressures than in the general population. By removing these ‘noisy’ indicators, we ensured that the final LPI-v3s prioritizes scales with the highest internal consistency, resulting in a leaner but more reliable instrument.

A significant structural finding was the emergence of Sensation-Seeking as a standalone dimension, independent of the broader Extraversion factor. While traditionally nested within Extraversion in the HEXACO model, its separation in this sample suggests that for athletes, the drive for physical risk and sensory stimulation is psychometrically distinct from social vitality. Similarly, while the broader Honesty–Humility domain was excluded due to insufficient structural stability, likely influenced by impression management or selection biases inherent in competitive sport, the H2: Greed-Avoidance facet was robustly retained. The items remaining in this factor (e.g., J46, J28, J40) show high factor loadings and low uniqueness, ensuring that the altruistic-prosocial axis of the HEXACO-based framework is still represented.

The retention of these two specific facets (Sensation-Seeking and Greed-Avoidance) alongside the five broad domains ensures that the LPI-v3s captures critical motivational and prosocial nuances that would otherwise be lost in a more reductive model. This result does not suggest that *Honesty–Humility* is irrelevant in sport; rather, it indicates that competitive environments may encourage impression management or socially desirable responding, restricting variability on self-report honesty items. Consequently, the LPI-v3s should be viewed as an empirically derived instrument optimized for personality dimensions that demonstrate stable measurement properties in the present athletic context. Future research may explore informant-based approaches to further assess honesty-related traits in sport populations.

### 4.4. Cultural and contextual considerations

Psychometric research has consistently shown that personality measures developed in one linguistic or cultural context may not function equivalently when applied elsewhere [7,16]. This issue is particularly relevant in smaller language communities such as Latvia, where adaptations of widely used instruments (e.g., the NEO-PI-R and Big Five Inventory) have demonstrated limitations in cultural sensitivity and interpretive precision [21,50]. The present study contributes to this body of work by empirically examining the measurement functioning of a locally developed instrument within a specific athletic context. While the findings are necessarily context-bound, they highlight the importance of evaluating personality measures within the cultural and applied settings in which they are intended to be used, rather than assuming cross-contextual equivalence.

The findings of Study 2 should be interpreted as evidence of exploratory criterion-related associations rather than as evidence of practical diagnostic accuracy. Although selected trait-level variables, particularly Anxious-Insecurity, Sociability, and Orderliness, were associated with elite/pre-elite status, the overall predictive performance was modest (AUC = 0.64). Therefore, the LPI-v3s should not be used as a diagnostic, selection, or talent-identification tool for determining athlete potential or competitive status. Its current utility is better understood as research-oriented and exploratory: it may provide structured information about personality variation in Latvian-speaking athlete samples, but any applied interpretation should be made cautiously and only as one component of a broader psychological assessment process that also includes contextual, developmental, sport-specific, and practitioner-based information.

### 4.5. Limitations

Several limitations of the present study should be acknowledged. First, although the shortened version of the LPI-v3s was developed using established psychometric procedures, the scale-reduction process relied on exploratory and confirmatory factor analyses conducted within the same stratified normative sample. The absence of an independent validation sample, split-sample cross-validation, or external replication limits confidence in the generalizability of the resulting factor structure. The iterative model refinement process may have increased the risk of sample-specific optimization; therefore, the retained structure should be interpreted as provisional rather than definitively confirmed. This methodological choice was made to preserve sufficient statistical power for measurement invariance testing and norm development across gender and age groups. Nevertheless, future studies should replicate the measurement model and normative findings in independent athlete samples before the LPI-v3s is used for confirmatory assessment purposes.

Second, the study relied exclusively on self-report data, which are inherently susceptible to response biases such as social desirability, impression management, and subjective self-perception. Although the inclusion of a Lie scale allowed for the identification and exclusion of potentially invalid response patterns, self-report instruments cannot fully eliminate such biases. Future research would benefit from incorporating multi-method assessment approaches, including informant ratings, behavioral indicators, or objective psychological measures, to strengthen construct validity.

Third, the cross-sectional design of the study precludes examination of developmental change and limits causal interpretation of associations between personality traits and sport-related variables. Age-related differences and normative patterns identified in this study should therefore be interpreted as cross-sectional comparisons rather than developmental trajectories. Longitudinal designs are needed to examine personality development in athletic populations over time.

Finally, the generalizability of the findings is constrained by the cultural and linguistic specificity of the sample. The stratified equal-cell norming strategy prioritized statistical balance across demographic strata rather than population-proportional representation, which may further limit the direct generalizability of normative distributions to the natural structure of athlete populations. The psychometric properties and normative reference values established in this study apply to Latvian-speaking athletes and may not directly generalize to athletes from other cultural or linguistic backgrounds. Cross-cultural replication and measurement equivalence testing in other athletic populations are necessary to evaluate broader applicability. Future studies should prioritize cross-validation of the LPI-v3s structure in independent athlete samples or alternative sport populations to evaluate the stability and generalizability of the refined model.

## 5. Conclusions

This study examined the psychometric functioning of a shortened version of the Latvian Personality Inventory (LPI-v3s) within an athlete population. The findings provide evidence supporting the internal consistency, interpretable factor structure, and gender-invariant measurement properties of the abbreviated instrument in this specific context. The development of age- and gender-specific reference values further highlight the importance of contextualized score interpretation when assessing personality traits in sport. The LPI-v3s represents a time-efficient, empirically evaluated personality measure whose current use is most appropriate for research and exploratory assessment contexts involving Latvian-speaking athletes. Given the provisional factorial structure and modest predictive performance, it should not be interpreted as a diagnostic, selection, or talent-identification tool. Future research should seek to replicate the measurement model in independent samples, examine longitudinal stability, and further explore criterion-related associations and cross-cultural applicability in other athletic populations.

## Supporting information

S1 Appendix Supplementary Tables. (DOCX)

S2 Appendix LPI-v3s Manual. (DOCX)

S3 Appendix Continuous Norm Tables. (XLSX)
