# Supplementary material for: Psychometric properties and standardization of the shortened latvian personality inventory (LPI-v3s) in athlete sample: Implications for evidence-based assessment
Source: PLoS One. 2026 Jul 22;21(7):e0352794. doi: 10.1371/journal.pone.0352794 (PMC13390810; doi:10.1371/journal.pone.0352794)
Supplement: S1 Appendix — (DOCX) [file pone.0352794.s001.docx]

**Appendix A. Supplementary Tables**

**Table S1.** **Composition of the normative sample by stratum: Initial counts and final selection**

| **Stratum**  *(gender, age, team vs individual sport)* | **Initial number of participants** | **Normative sample** | | |
| --- | --- | --- | --- | --- |
|  |  | *Number of participants* | *Mean age (years)  M (SD)* | *Age range (Years)*  *[min; max]* |
| Male 15-17, team sport | 105 | 32 | 15.7 (0.99) | [15;17] |
| Male 15-17, individual sport | 33 | 32 | 16.4 (0.67) | [15;17] |
| Male 18-20, team sport | 138 | 32 | 19.0 (0.74) | [18;20] |
| Male 18-20, individual sport | 60 | 32 | 19.3 (0.79) | [18;20] |
| Male 21-29, team sport | 92 | 32 | 23.0 (2.27) | [21;29] |
| Male 21-29, individual sport | 64 | 32 | 23.0 (1.84) | [21;28] |
| Male 30-45, team sport | 21 | 21 | 37.3 (4.59) | [30;44] |
| Male 30-45, individual sport | 22 | 21 | 37.2 (5.12) | [30;44] |
| Total male, team sport | 356 | 117 | 22.5 (7.82) | [15;44] |
| Total male, individual Sport | 179 | 117 | 22.8 (7.61) | [15;44] |
| **Total male** | **535** | **234** | **22.6 (7.70)** | **[15;44]** |
| Female 15-17, team sport | 32 | 32 | 15.7 (1.30) | [15;17] |
| Female 15-17, individual sport | 37 | 32 | 16.4 (0.71) | [15;17] |
| Female 18-20, team sport | 50 | 32 | 19.2 (0.72) | [18;20] |
| Female 18-20, individual sport | 84 | 32 | 19.2 (0.71) | [18;20] |
| Female 21-29, team sport | 47 | 32 | 22.3 (1.59) | [21;28] |
| Female 21-29, individual sport | 119 | 32 | 23.1 (2.03) | [21;29] |
| Female 30-45, team sport | 5 | 5 | 36.6 (6.50) | [30;43] |
| Female 30-45, individual sport | 16 | 5 | 36.4 (5.08) | [30;43] |
| Total female, team sport | 134 | 101 | 19.9 (4.96) | [15;43] |
| Total female, individual sport | 256 | 101 | 20.4 (4.82) | [15;43] |
| **Total female** | **390** | **202** | **20.2 (4.89)** | **[15;43]** |
| **Total** | **925** | **436** | **21.5 (6.66)** | **[15;44]** |

**Table S2.** **Participant distributes in the normative sample by age group and gender**

| **Performance level** | **Gender** | **Count** | **% of total normative sample** |
| --- | --- | --- | --- |
| Adolescents/Young adults (15–20 years) (*n*=256) | Male | 128 | 29.4% |
|  | Female | 128 | 29.4% |
| Adults (21–45 years) (*n*=180) | Male | 106 | 24.3% |
|  | Female | 74 | 17.0% |

**Table S3.** **Participant distribution in the analysis sample by sport type, performance level and gender**

| **Sport type** | **Performance level** | **Gender** | **Counts** | | **% of total** |
| --- | --- | --- | --- | --- | --- |
| Team  (*n*=391) | Non-elite (*n*=184) | Male | | 136 | 18.1% |
|  |  | Female | | 48 | 6.4% |
|  | Pre-elite (*n*=199) | Male | | 147 | 19.5% |
|  |  | Female | | 52 | 6.9% |
|  | Elite (*n*=8) | Male | | 6 | 0.8% |
|  |  | Female | | **2** | 0.4% |
| Individual  (*n*=361) | Non-elite (*n*=163) | Male | | 58 | 7.7% |
|  |  | Female | | 105 | 13.9% |
|  | Pre-elite (*n*=172) | Male | | 81 | 10.8% |
|  |  | Female | | 91 | 12.1% |
|  | Elite (*n*=26) | Male | | 9 | 1.2% |
|  |  | Female | | 17 | 2.3% |

**Table S4. Goodness-of-fit indices of HCFA models**

| **Model** | **Sample** | **N** | **χ^2^ (df)** | **p** | **CFI** | **TLI** | **RMSEA [90% CI]** | **SRMR** |
| --- | --- | --- | --- | --- | --- | --- | --- | --- |
| *Initial HCFA Model* | Normative sample | 436 | 7484 (4424) | <0.001 | 0.728 | 0.719 | 0.040  [0.038; 0.041] | 0.087 |
| *Modified Model 1* | Normative sample | 436 | 6582 (3695) | <0.001 | 0.748 | 0.739 | 0.042  [0.041; 0.044] | 0.087 |
| *Modified Model 2* | Normative sample | 436 | 6474 (3609) | <0.001 | 0.750 | 0.741 | 0.043  [0.041; 0.044] | 0.087 |
| *Modified Model 3* | Normative sample | 436 | 4979 (2738) | <0.001 | 0.799 | 0.791 | 0.043  [0.041; 0.045] | 0.084 |
| *Modified Model 4* | Normative sample | 436 | 4540 (2642) | <0.001 | 0.830 | 0.822 | 0.041  [0.039; 0.043] | 0.077 |
| *Modified Model 4* | Male  athletes | 234 | 3700 (2642) | <0.001 | 0.783 | 0.772 | 0.041  [0.038; 0.045] | 0.093 |
| *Modified Model 4* | Female athletes | 202 | 3373 (2642) | <0.001 | 0.832 | 0.824 | 0.037  [0.033; 0.041] | 0.091 |

*Note.* Estimator is DWLS. Model test is scaled and shifted. Information matrix is expected. Standard errors are robust. Fit indices are based on the scaled test statistics. χ²/df: Degrees of freedom; CFI: Confirmatory Fit Index; TLI: Tucker-Lewis Index; RMSEA: Root Mean Square; SRMR: Standardized Root Mean Square Residual

**Table S5. Exploratory factor analysis (principal axis factoring) of the LPI-v3s: Factor loadings for the 15-factor solution using promax rotation**

| **Item** | **Factor 1** | **Factor 2** | **Factor 3** | **Factor 4** | **Factor 5** | **Factor**  **6** | **Factor 7** | **Factor 8** | **Factor 9** | **Factor 10** | **Factor 11** | **Factor 12** | **Factor 13** | **Factor 14** | **Factor 15** | **Uniqueness** |
| --- | --- | --- | --- | --- | --- | --- | --- | --- | --- | --- | --- | --- | --- | --- | --- | --- |
| J33 | 0.70 |  |  |  |  |  |  |  |  |  |  |  |  |  |  | 0.42 |
| J27 | -0.69 |  |  |  |  |  |  |  |  |  |  |  |  |  |  | 0.36 |
| J58 | 0.68 |  |  |  |  |  |  |  |  |  |  |  |  |  |  | 0.25 |
| J70 | 0.66 |  |  |  |  |  |  |  |  |  |  |  |  |  |  | 0.30 |
| J45 | 0.64 |  |  |  |  |  |  |  |  |  |  |  |  |  |  | 0.39 |
| J39 | -0.61 |  |  |  |  |  |  |  |  |  |  |  |  |  |  | 0.34 |
| J77 | 0.59 |  |  |  |  |  |  |  |  |  |  |  |  |  |  | 0.44 |
| J14 | 0.56 |  |  |  |  |  |  |  |  |  |  |  |  |  |  | 0.55 |
| J90 | 0.52 |  |  |  |  |  |  |  |  |  |  |  |  |  |  | 0.54 |
| J7 |  | 0.89 |  |  |  |  |  |  |  |  |  |  |  |  |  | 0.21 |
| J19 |  | 0.81 |  |  |  |  |  |  |  |  |  |  |  |  |  | 0.36 |
| J13 |  | -0.56 |  |  |  |  |  |  |  |  |  |  |  |  |  | 0.46 |
| J1 |  | 0.54 |  |  |  |  |  |  |  |  |  |  |  |  |  | 0.56 |
| J56 |  |  | 0.78 |  |  |  |  |  |  |  |  |  |  |  |  | 0.33 |
| J74 |  |  | 0.72 |  |  |  |  |  |  |  |  |  |  |  |  | 0.36 |
| J68 |  |  | 0.66 |  |  |  |  |  |  |  |  |  |  |  |  | 0.53 |
| J62 |  |  | 0.51 |  |  |  |  |  |  |  |  |  |  |  |  | 0.51 |
| J80 |  |  |  | 0.79 |  |  |  |  |  |  |  |  |  |  |  | 0.25 |
| J87 |  |  |  | -0.66 |  |  |  |  |  |  |  |  |  |  |  | 0.43 |
| J93 |  |  |  | 0.63 |  |  |  |  |  |  |  |  |  |  |  | 0.40 |
| J99 |  |  |  | 0.61 |  |  |  |  |  |  |  |  |  |  |  | 0.47 |
| J48 |  |  |  |  | 0.75 |  |  |  |  |  |  |  |  |  |  | 0.39 |
| J42 |  |  |  |  | 0.70 |  |  |  |  |  |  |  |  |  |  | 0.46 |
| J36 |  |  |  |  | 0.63 |  |  |  |  |  |  |  |  |  |  | 0.51 |
| J97 |  |  |  |  | 0.53 |  |  |  |  |  |  |  |  |  |  | 0.65 |
| J54 |  |  |  |  |  | 0.79 |  |  |  |  |  |  |  |  |  | 0.39 |
| J66 |  |  |  |  |  | 0.75 |  |  |  |  |  |  |  |  |  | 0.33 |
| J72 |  |  |  |  |  | 0.56 |  |  |  |  |  |  |  |  |  | 0.65 |
| J60 |  |  |  |  |  | -0.47 |  |  |  |  |  |  |  |  |  | 0.58 |
| J29 |  |  |  |  |  |  | 0.75 |  |  |  |  |  |  |  |  | 0.38 |
| J35 |  |  |  |  |  |  | 0.67 |  |  |  |  |  |  |  |  | 0.44 |
| J47 |  |  |  |  |  |  | 0.64 |  |  |  |  |  |  |  |  | 0.46 |
| J10 |  |  |  |  |  |  |  | -0.75 |  |  |  |  |  |  |  | 0.35 |
| J16 |  |  |  |  |  |  |  | 0.74 |  |  |  |  |  |  |  | 0.46 |
| J4 |  |  |  |  |  |  |  | 0.72 |  |  |  |  |  |  |  | 0.41 |
| J50 |  |  |  |  |  |  |  |  | 0.61 |  |  |  |  |  |  | 0.50 |
| J75 |  |  |  |  |  |  |  |  | 0.55 |  |  |  |  |  |  | 0.64 |
| J82 |  |  |  |  |  |  |  |  | 0.47 |  |  |  |  |  |  | 0.62 |
| J25 |  |  |  |  |  |  |  |  | 0.46 |  |  |  |  |  |  | 0.65 |
| J18 |  |  |  |  |  |  |  |  |  | 0.68 |  |  |  |  |  | 0.44 |
| J24 |  |  |  |  |  |  |  |  |  | -0.64 |  |  |  |  |  | 0.48 |
| J12 |  |  |  |  |  |  |  |  |  | 0.62 |  |  |  |  |  | 0.60 |
| J76 |  |  |  |  |  |  |  |  |  |  | 0.66 |  |  |  |  | 0.44 |
| J89 |  |  |  |  |  |  |  |  |  |  | 0.65 |  |  |  |  | 0.47 |
| J95 |  |  |  |  |  |  |  |  |  |  | 0.42 |  |  |  |  | 0.54 |
| J81 |  |  |  |  |  |  |  |  |  |  |  | 0.73 |  |  |  | 0.36 |
| J94 |  |  |  |  |  |  |  |  |  |  |  | -0.62 |  |  |  | 0.49 |
| J88 |  |  |  |  |  |  |  |  |  |  |  | 0.45 |  |  |  | 0.57 |
| J26 |  |  |  |  |  |  |  |  |  |  |  |  | 0.62 |  |  | 0.56 |
| J32 |  |  |  |  |  |  |  |  |  |  |  |  | -0.56 |  |  | 0.49 |
| J44 |  |  |  |  |  |  |  |  |  |  |  |  | 0.56 |  |  | 0.42 |
| J46 |  |  |  |  |  |  |  |  |  |  |  |  |  | -0.78 |  | 0.38 |
| J28 |  |  |  |  |  |  |  |  |  |  |  |  |  | 0.58 |  | 0.58 |
| J40 |  |  |  |  |  |  |  |  |  |  |  |  |  | 0.53 |  | 0.58 |
| J61 |  |  |  |  |  |  |  |  |  |  |  |  |  |  | 0.57 | 0.61 |
| J55 |  |  |  |  |  |  |  |  |  |  |  |  |  |  | -0.56 | 0.51 |
| J67 |  |  |  |  |  |  |  |  |  |  |  |  |  |  | -0.35 | 0.64 |
| SumSq. Loadings | 4.20 | 2.51 | 2.49 | 2.39 | 2.37 | 1.98 | 1.94 | 1.80 | 1.75 | 1.60 | 1.60 | 1.56 | 1.56 | 1.47 | 1.39 |  |
| Proportion var. | 0.07 | 0.04 | 0.04 | 0.04 | 0.04 | 0.04 | 0.03 | 0.03 | 0.03 | 0.03 | 0.03 | 0.03 | 0.03 | 0.03 | 0.02 |  |
| Cronbah's alfa | 0.84 | 0.78 | 0.73 | 0.79 | 0.70 | 0.69 | 0.73 | 0.73 | 0.56 | 0.62 | 0.64 | 0.63 | 0.63 | 0.62 | 0.57 |  |

*Note*. *N* = 436. The extraction method was Principal Axis Factoring with Promax rotation. Kaiser-Meyer-Olkin (KMO) measure of sampling adequacy = 0.749. Total variance explained = 53.7%. Factor 1 = N1: Anxious-Insecurity (includes items from original N1–N4 facets); Factor 2 = C1: Orderliness; Factor 3 = O3: Inquisitiveness; Factor 4 = A4: Composure; Factor 5 = A2: Gentleness; Factor 6 = E3: Sensation-Seeking; Factor 7 = E2: Joyfulness; Factor 8 = E1: Sociability; Factor 9 = M: Lie Scale; Factor 10 = O1: Aesthetic Interests; Factor 11 = C4: Prudence; Factor 12 = O4: Creativity; Factor 13 = C2: Self-discipline; Factor 14 = H2: Greed-Avoidance; Factor 15 = A3: Obedience. Factor loadings < 0.34 are not presented.

**Table S6.** **Second-order exploratory factor analysis of the LPI-v3s facets: Factor loadings using principal axis factoring and varimax rotation**

| **Scale** | **Factor 1** | **Factor 2** | **Factor 3** | **Factor 4** | **Factor 5** | **Factor 6** | **Uniqueness** |
| --- | --- | --- | --- | --- | --- | --- | --- |
| C1 | 0.75 |  |  |  |  |  | 0.32 |
| C2 | 0.57 |  |  |  |  |  | 0.57 |
| C4 | 0.55 |  |  |  |  | 0.32 | 0.56 |
| A2 |  | 0.64 |  |  |  |  | 0.57 |
| A3 |  | 0.60 |  |  |  |  | 0.61 |
| A4 |  | 0.47 | -0.55 |  |  |  | 0.43 |
| N1_new |  |  | 0.75 |  |  |  | 0.34 |
| O3 |  |  |  | 0.59 |  | 0.42 | 0.38 |
| O1 |  |  |  | 0.53 |  |  | 0.71 |
| O4 |  |  |  | 0.49 | 0.33 |  | 0.60 |
| E2 |  |  |  |  | 0.65 |  | 0.48 |
| E1 |  |  |  |  | 0.53 |  | 0.72 |
| E3 |  |  |  |  |  | -0.35 | 0.71 |
| H2 |  |  |  |  |  |  | 0.90 |
| SumSq. Loadings | 1.34 | 1.16 | 1.05 | 0.99 | 0.97 | 0.61 |  |
| Proportion var. | 0.1 | 0.08 | 0.08 | 0.07 | 0.07 | 0.04 |  |

*Note*. *N* = 436. The extraction method was Principal Axis Factoring with Varimax rotation. Number of factors was determined based on Parallel Analysis. Decomposition was based on the correlation matrix (interval-level data). Total variance explained = 43.7%. Factor loadings < 0.34 are not presented; loadings > 0.40 are shown in bold. Factor 1 = Conscientiousness; Factor 2 = Agreeableness; Factor 3 = Neuroticism; Factor 4 = Openness; Factor 5 = Extraversion; Factor 6 = Uninterpretable. The A4: Composure scale demonstrated significant loadings on both Factor 3 (when reversed as N2: Irritability) and Factor 2 (Agreeableness). The E3: Sensation-seeking scale did not meet the criteria for inclusion in the five-factor domain structure.

**Table S7**. **Pearson correlations between LPI-v3s scales and age in the normative sample**

| **LPI-v3s scale** | **Pearson correlation with age** | | |
| --- | --- | --- | --- |
|  | *Total normative sample (n=436)* | *Female normative subsample (n=202)* | *Male normative subsample (n=234)* |
| N1_new: Anxious-Insecurity | -0.12* | -0.18** | -0.01 |
| N2_new: Irritability | -0.03 | -0.08 | 0.07 |
| E1: Sociability | 0.04 | 0.00 | 0.09 |
| E2: Joyfulness | -0.01 | -0.01 | -0.02 |
| E3: Sensation-Seeking | -0.14** | -0.19** | -0.12 |
| C1: Orderliness | 0.10* | 0.19** | 0.07 |
| C2: Self-discipline | 0.11* | 0.10 | 0.08 |
| C4: Prudence | 0.20*** | 0.14* | 0.22*** |
| A2: Gentleness | -0.06 | 0.00 | -0.09 |
| A3: Obedience | -0.13** | -0.01 | -0.18** |
| A4: Composure | 0.03 | 0.08 | -0.07 |
| O1: Aesthetic Interests | -0.04 | -0.07 | 0.04 |
| O3: Inquisitiveness | 0.20*** | 0.04 | 0.28*** |
| O4: Creativity | 0.07 | -0.01 | 0.11 |
| H2: Greed-Avoidance | 0.18*** | 0.02 | 0.31*** |
| N: Neuroticism | -0.10* | -0.18* | 0.02 |
| E: Extraversion | 0.03 | 0.00 | 0.05 |
| C: Conscientiousness | 0.17*** | 0.20** | 0.15* |
| A: Agreeableness | -0.08 | 0.02 | -0.17** |
| O: Openness to Experience | 0.11* | -0.02 | 0.23*** |
| M: Lie Scale | -0.01 | 0.02 | -0.02 |

*Note*. * *p* <0.05, ** *p* <0.01, *** *p* <0.001.

**Table S8.** **Mean differences and effect sizes between age groups in the female subsample (adolescents/young adults [A/YA, 15–20] vs. adults [21–45])**

| **LPI-v3 Scale (short form)** | **Mean (*SD*) A/YA (*n*=128)** | **Mean (*SD*) adults (*n*=74)** | **Test**  **used** | **Test statistic** | ***p*-value** | **Effect size**  **d / r** | **Norms required** |
| --- | --- | --- | --- | --- | --- | --- | --- |
| N: Neuroticism | 30.0 (6.42) | 26.8 (6.84) | T-test | t (200) = 3.35 | <0.001 | 0.49 | Yes |
| N1_new: Anxious-Insecurity | 29.4 (7.43) | 26.4 (7.11) | T-test | t (200) = 2.79 | 0.006 | 0.41 | Yes |
| C: Conscientiousness | 31.5 (5.67) | 34.1 (6.75) | T-test | t (200) = -2.97 | <0.001 | -0.43 | Yes |
| C1: Orderliness | 33.0 (7.89) | 35.9 (8.62) | Mann-Whitney U | U = 3775 | 0.013 | *r*=0.20 | Yes |
| C4: Prudence | 31.0 (7.08) | 33.3 (8.34) | T-test | t (200) = -2.11 | 0.037 | -0.31 | Yes |
| E3: Sensation-Seeking | 34.8 (8.77) | 33.4 (9.14) | Mann-Whitney U | U=4348 | 0.033 | *r*=-0.08 | No |

*Note*. Hₐ μ15-20 years ≠ μ21-45 years. Cohen's (Effect size for -tests) and Rank Biserial Correlation (*r*) (Effect size for Mann-Whitney tests) are reported. Norms Required indicates scales that met both the correlation criterion (*p* <.05 with age) and the effect size criterion (*d* or *r* ≥ 0.20).

**Table S9.** **Mean differences and effect sizes between age groups in male subsample (adolescents/young adults [A/YA, 15–20] vs. Adults [21–45])**

| **LPI-v3 Scale**  **(short form)** | **Mean (SD) A/YA (*n*=128)** | **Mean**  **(SD)**  **adults (*n*=106)** | **Test used** | **Test statistic** | ***p*-value** | **Effect Size**  **d / r** | **Norms required** |
| --- | --- | --- | --- | --- | --- | --- | --- |
| C: Conscientiousness | 32.2 (6.58) | 34.4 (6.24) | T-test | t (232) = -2.60 | 0.010 | -0.34 | Yes |
| C4: Prudence | 30.8 (7.88) | 34.4 (8.41) | Mann-Whitney U | U = 5167 | <0.001 | *r*=0.24 | Yes |
| A: Agreeableness | 33.5 (6.10) | 32.1 (6.71) | T-test | t (232) = 1.64 | 0.103 | 0.21 | No |
| A3: Obedience | 33.6 (7.27) | 29.8 (8.43) | Mann-Whitney U | U = 4920 | <0.001 | *r*=-0.27 | Yes |
| O: Openness to Experience | 29.1 (5.19) | 30.6 (5.64) | T-test | t (232) = -2.14 | 0.034 | -0.28 | Yes |
| O3: Inquisitiveness | 31.4 (7.91) | 34.0 (9.45) | Mann-Whitney U | U = 4645 | 0.014 | *r*=0.19 | No |
| H2: Greed-Avoidance | 27.9 (8.77) | 32.7 (9.14) | Mann-Whitney U | U = 4348 | <0.001 | *r*=0.32 | Yes |

*Note*. Hₐ μ15-20 years ≠ μ21-45 years. Cohen's (Effect size for -tests) and Rank Biserial Correlation (*r*) (Effect size for Mann-Whitney tests) are reported. Norms Required indicates scales that met both the correlation criterion (*p* <.05 with age) and the effect size criterion (*d* or *r* ≥ 0.20).
