# Supplementary material for: Psychometric properties and standardization of the shortened latvian personality inventory (LPI-v3s) in athlete sample: Implications for evidence-based assessment
Source: PLoS One. 2026 Jul 22;21(7):e0352794. doi: 10.1371/journal.pone.0352794 (PMC13390810; doi:10.1371/journal.pone.0352794)
Supplement: S2 Appendix — (DOCX) [file pone.0352794.s002.docx]

**Appendix B. LPI-v3s Manual (English and Latvian)**

**Latvian Personality Inventory (LPI-v3s):** **Norms for Athletes**

Self-description form

***Viktorija Perepjolkina, Viesturs Reņģe, 2013; Viktorija Perepjolkina, 2025.***

*Please read each statement carefully and rate how well it describes you or reflects your opinion.. There are no right or wrong answers here!*

*Please describe yourself honestly, as you are now, not as you would like to be. Next to EACH statement, mark one of the numbers that most accurately describes your opinion. Use this scale for your answers:*

| **1** | **2** | **3** | **4** | **5** |
| --- | --- | --- | --- | --- |
| **does not match** | **rather does not match** | **can't decide** | **rather matches** | **matches** |

| 1. (1.) | I am a very punctual person. | 1 | 2 | 3 | 4 | 5 |
| --- | --- | --- | --- | --- | --- | --- |
| 2. (4.) | I like to be around a lot of people. | 1 | 2 | 3 | 4 | 5 |
| 3. (7.) | I maintain almost perfect order at home. | 1 | 2 | 3 | 4 | 5 |
| 4. (10.) | I feel better alone than among people. | 1 | 2 | 3 | 4 | 5 |
| 5. (12.) | I am a fan of opera or ballet. | 1 | 2 | 3 | 4 | 5 |
| 6. (13.) | I tend to "lay out" my belongings, not putting them in the right places. | 1 | 2 | 3 | 4 | 5 |
| 7. (14.) | I am often troubled by thoughts that something bad might happen. | 1 | 2 | 3 | 4 | 5 |
| 8. (16.) | I can't go long without the company of other people. | 1 | 2 | 3 | 4 | 5 |
| 9. (18.) | I am a connoisseur of a certain art genre. | 1 | 2 | 3 | 4 | 5 |
| 10. (19.) | I have an inner need to keep everything clean and tidy. | 1 | 2 | 3 | 4 | 5 |
| 11. (24.) | Art has no special meaning in my life. | 1 | 2 | 3 | 4 | 5 |
| 12. (25.) | I have never had the desire to take revenge on my abuser. | 1 | 2 | 3 | 4 | 5 |
| 13. (26.) | I do what I have planned, regardless of my mood or desire to do it. | 1 | 2 | 3 | 4 | 5 |
| 14. (27.) | I have the ability to work successfully under stress. | 1 | 2 | 3 | 4 | 5 |
| 15. (28.) | I am indifferent to expensive luxury goods. | 1 | 2 | 3 | 4 | 5 |
| 16. (29.) | I have an easy, cheerful personality. | 1 | 2 | 3 | 4 | 5 |
| 17. (32.) | I often leave unpleasant or difficult tasks until the last minute. | 1 | 2 | 3 | 4 | 5 |
| 18. (33.) | When problems arise, I often get confused and don't know what to do. | 1 | 2 | 3 | 4 | 5 |
| 19. (35.) | I am a cheerful person by nature. | 1 | 2 | 3 | 4 | 5 |
| 20. (36.) | I tend to be sarcastic. | 1 | 2 | 3 | 4 | 5 |
| 21. (39.) | I keep a cool head in stressful situations. | 1 | 2 | 3 | 4 | 5 |
| 22. (40.) | A large amount of money has no special meaning in my life. | 1 | 2 | 3 | 4 | 5 |
| 23. (42.) | Some say I have a "sharp tongue." | 1 | 2 | 3 | 4 | 5 |
| 24. (44.) | I know how to plan my time so that I have time to do everything I need to do. | 1 | 2 | 3 | 4 | 5 |
| 25. (45). | When faced with difficulties, I quickly lost courage and faith in myself. | 1 | 2 | 3 | 4 | 5 |
| 26. (46.) | I would like to have a lot of expensive brand-name things. | 1 | 2 | 3 | 4 | 5 |
| 27. (47.) | It is easy for me to create a happy and joyful mood around myself. | 1 | 2 | 3 | 4 | 5 |
| 28. (48.) | I tend to be angry with others. | 1 | 2 | 3 | 4 | 5 |
| 29. (50.) | There is no one I would think badly of. | 1 | 2 | 3 | 4 | 5 |
| 30. (54.) | I like activities that give you adrenaline and thrills. | 1 | 2 | 3 | 4 | 5 |
| 31. (55.) | It's hard for me to be obedient. | 1 | 2 | 3 | 4 | 5 |
| 32. (56.) | I have a thirst for knowledge. | 1 | 2 | 3 | 4 | 5 |
| 33. (58.) | I have a thirst for knowledge. | 1 | 2 | 3 | 4 | 5 |

| **1** | | **2** | **3** | **4** | **5** | | | | | |
| --- | --- | --- | --- | --- | --- | --- | --- | --- | --- | --- |
| **does not match** | | **rather does not match** | **can't decide** | **rather matches** | **matches** | | | | | |
| 34. (60.) | I don't do anything dangerous for fun. | | | | | 1 | 2 | 3 | 4 | 5 |
| 35. (61.) | I will unconditionally carry out the instructions of my superiors. | | | | | 1 | 2 | 3 | 4 | 5 |
| 36. (62.) | I enjoy reading scientific literature. | | | | | 1 | 2 | 3 | 4 | 5 |
| 37. (66.) | I like to take risks. | | | | | 1 | 2 | 3 | 4 | 5 |
| 38. (67.) | I have difficulty submitting to the will of others. | | | | | 1 | 2 | 3 | 4 | 5 |
| 39. (68.) | I like intellectual challenges. | | | | | 1 | 2 | 3 | 4 | 5 |
| 40. (70.) | I often feel sad. | | | | | 1 | 2 | 3 | 4 | 5 |
| 41. (72.) | I believe that you should try everything at least once in your life. | | | | | 1 | 2 | 3 | 4 | 5 |
| 42. (74.) | I am curious, I want to learn and explore everything. | | | | | 1 | 2 | 3 | 4 | 5 |
| 43. (75.) | I never talk down to other people. | | | | | 1 | 2 | 3 | 4 | 5 |
| 44. (76.) | I usually prepare myself carefully before I take action. | | | | | 1 | 2 | 3 | 4 | 5 |
| 45. (77.) | I worry about what people around me think of me. | | | | | 1 | 2 | 3 | 4 | 5 |
| 46. (80.) | I can get irritated quickly. | | | | | 1 | 2 | 3 | 4 | 5 |
| 47. (81.) | I often come up with innovative, original ideas. | | | | | 1 | 2 | 3 | 4 | 5 |
| 48. (82.) | I like every person I meet. | | | | | 1 | 2 | 3 | 4 | 5 |
| 49. (87.) | It's pretty hard to make me angry. | | | | | 1 | 2 | 3 | 4 | 5 |
| 50. (88.) | I often find unusual uses for ordinary things. | | | | | 1 | 2 | 3 | 4 | 5 |
| 51. (89.) | Before I do anything, I carefully consider the possible consequences. | | | | | 1 | 2 | 3 | 4 | 5 |
| 52. (90.) | I usually take criticism painfully. | | | | | 1 | 2 | 3 | 4 | 5 |
| 53. (93.) | I can get angry even over little things. | | | | | 1 | 2 | 3 | 4 | 5 |
| 54. (94.) | I could hardly be called a creative person. | | | | | 1 | 2 | 3 | 4 | 5 |
| 55. (95.) | I love to carefully plan even the smallest activities and work matters. | | | | | 1 | 2 | 3 | 4 | 5 |
| 56. (97.) | I wouldn't feel guilty about getting mad at someone I don't like. | | | | | 1 | 2 | 3 | 4 | 5 |
| 57. (99.) | When I get angry, I quickly lose my temper. | | | | | 1 | 2 | 3 | 4 | 5 |

**Additional information: ________________________________________________________________**

**____________________________________________________________________________________**

**____________________________________________________________________________________**

**____________________________________________________________________________________**

*Note.* The English translation of the Latvian Personality Inventory (LPI-v3) is provided for reference to aid comprehension by non-Latvian readers. The original validated version is in Latvian. This English version has not undergone psychometric validation and should not be used for data collection.

**Latvijas Personības aptauja (LPA-v3s) normas sportistiem**Pašraksturojuma forma

***Viktorija Perepjolkina, Viesturs Reņģe, 2013; Viktorija Perepjolkina, 2025.***

*Lūdzu, rūpīgi izlasiet katru apgalvojumu un novērtējiet, cik lielā mērā katrs no tiem raksturo Jūs vai atbilst Jūsu viedoklim. Šeit nav pareizu vai nepareizu atbilžu!
Lūdzu, raksturojiet sevi atklāti, tādu, kāds/kāda esat šobrīd, nevis kāds/kāda vēlaties būt. Godīgas atbildes ļaus Jums iegūt precīzāku savas personības raksturojumu. Pretī KATRAM apgalvojumam atzīmējiet vienu no cipariem, kas visprecīzāk raksturo Jūsu viedokli. Atbildēm izmantojiet šo skalu:*

| **1** | **2** | **3** | **4** | **5** |
| --- | --- | --- | --- | --- |
| **neatbilst** | **drīzāk neatbilst** | **nevaru izlemt** | **drīzāk atbilst** | **atbilst** |

| 1. (1.) | Es esmu ļoti akurāts cilvēks. | 1 | 2 | 3 | 4 | 5 |
| --- | --- | --- | --- | --- | --- | --- |
| 2. (4.) | Man patīk, ja apkārt ir daudz cilvēku. | 1 | 2 | 3 | 4 | 5 |
| 3. (7.) | Mājās es uzturu gandrīz ideālu kārtību. | 1 | 2 | 3 | 4 | 5 |
| 4. (10.) | Es labāk jūtos vienatnē nekā starp cilvēkiem. | 1 | 2 | 3 | 4 | 5 |
| 5. (12.) | Esmu operas vai baleta cienītājs. | 1 | 2 | 3 | 4 | 5 |
| 6. (13.) | Mēdzu „izmētāt” savas mantas, nenoliekot tās īstajās vietās. | 1 | 2 | 3 | 4 | 5 |
| 7. (14.) | Mani bieži nomāc domas, ka var notikt kas slikts. | 1 | 2 | 3 | 4 | 5 |
| 8. (16.) | Nevaru ilgi iztikt bez citu cilvēku sabiedrības. | 1 | 2 | 3 | 4 | 5 |
| 9. (18.) | Esmu kāda mākslas žanra pazinējs. | 1 | 2 | 3 | 4 | 5 |
| 10. (19.) | Man ir iekšēja nepieciešamība visu uzturēt tīrībā un kārtībā. | 1 | 2 | 3 | 4 | 5 |
| 11. (24.) | Mākslai nav īpašas nozīmes manā dzīvē. | 1 | 2 | 3 | 4 | 5 |
| 12. (25.) | Man nekad nav bijusi vēlme atriebties savam pāridarītājam. | 1 | 2 | 3 | 4 | 5 |
| 13. (26.) | Izdaru to, ko esmu ieplānojis, neatkarīgi no garastāvokļa vai vēlmes to darīt. | 1 | 2 | 3 | 4 | 5 |
| 14. (27.) | Spēju veiksmīgi darboties stresa apstākļos. | 1 | 2 | 3 | 4 | 5 |
| 15. (28.) | Esmu vienaldzīgs pret dārgām luksus precēm. | 1 | 2 | 3 | 4 | 5 |
| 16. (29.) | Man ir viegls, dzīvespriecīgs raksturs. | 1 | 2 | 3 | 4 | 5 |
| 17. (32.) | Bieži atstāju nepatīkama vai sarežģīta darba izpildi uz pēdējo brīdi. | 1 | 2 | 3 | 4 | 5 |
| 18. (33.) | Problēmu gadījumā es bieži apjūku un nezinu, ko iesākt. | 1 | 2 | 3 | 4 | 5 |
| 19. (35.) | Pēc dabas esmu jautrs cilvēks. | 1 | 2 | 3 | 4 | 5 |
| 20. (36.) | Mēdzu būt sarkastisks un dzēlīgs. | 1 | 2 | 3 | 4 | 5 |
| 21. (39.) | Stresa situācijā saglabāju vēsu prātu. | 1 | 2 | 3 | 4 | 5 |
| 22. (40.) | Lielam naudas daudzumam nav īpašas nozīmes manā dzīvē. | 1 | 2 | 3 | 4 | 5 |
| 23. (42.) | Daži saka, ka man esot „asa mēle”. | 1 | 2 | 3 | 4 | 5 |
| 24. (44.) | Es protu plānot savu laiku tā, lai pagūtu izdarīt visu nepieciešamo. | 1 | 2 | 3 | 4 | 5 |
| 25. (45). | Saskaroties ar sarežģījumiem, es ātri zaudēju drosmi un ticību sev. | 1 | 2 | 3 | 4 | 5 |
| 26. (46.) | Es gribētu, lai man ir daudz dārgo zīmolu lietu. | 1 | 2 | 3 | 4 | 5 |
| 27. (47.) | Man ir viegli radīt ap sevi priecīgu un līksmu noskaņojumu. | 1 | 2 | 3 | 4 | 5 |
| 28. (48.) | Mēdzu būt negants pret citiem. | 1 | 2 | 3 | 4 | 5 |
| 29. (50.) | Nav tāda cilvēka, par kuru es slikti domātu. | 1 | 2 | 3 | 4 | 5 |
| 30. (54.) | Man patīk nodarbes, kas dod adrenalīnu un asas izjūtas. | 1 | 2 | 3 | 4 | 5 |
| 31. (55.) | Man ir grūti būt paklausīgam. | 1 | 2 | 3 | 4 | 5 |
| 32. (56.) | Man piemīt tieksme pēc zināšanām. | 1 | 2 | 3 | 4 | 5 |
| 33. (58.) | Bieži jūtos bēdīgs un nomākts. | 1 | 2 | 3 | 4 | 5 |

| **1** | | **2** | **3** | **4** | | **5** | | | | |
| --- | --- | --- | --- | --- | --- | --- | --- | --- | --- | --- |
| **neatbilst** | | **drīzāk neatbilst** | **nevaru izlemt** | **drīzāk atbilst** | | **atbilst** | | | | |
| 34. (60.) | Nedaru neko bīstamu izpriecu dēļ. | | | | 1 | | 2 | 3 | 4 | 5 |
| 35. (61.) | Bez ierunām izpildu augstākstāvošo personu teikto. | | | | 1 | | 2 | 3 | 4 | 5 |
| 36. (62.) | Labprāt lasu zinātnisko literatūru. | | | | 1 | | 2 | 3 | 4 | 5 |
| 37. (66.) | Man patīk riskēt. | | | | 1 | | 2 | 3 | 4 | 5 |
| 38. (67.) | Ar grūtībām pakļaujos citu gribai. | | | | 1 | | 2 | 3 | 4 | 5 |
| 39. (68.) | Man patīk intelektuāli izaicinājumi. | | | | 1 | | 2 | 3 | 4 | 5 |
| 40. (70.) | Bieži jūtos noskumis. | | | | 1 | | 2 | 3 | 4 | 5 |
| 41. (72.) | Uzskatu, ka dzīvē kaut vienu reizi jāizmēģina itin viss. | | | | 1 | | 2 | 3 | 4 | 5 |
| 42. (74.) | Es esmu zinātkārs, gribu visu izzināt un izpētīt. | | | | 1 | | 2 | 3 | 4 | 5 |
| 43. (75.) | Es nekad neaprunāju citus cilvēkus. | | | | 1 | | 2 | 3 | 4 | 5 |
| 44. (76.) | Parasti rūpīgi sagatavojos, pirms sāku rīkoties. | | | | 1 | | 2 | 3 | 4 | 5 |
| 45. (77.) | Uztraucos par to, ko apkārtējie cilvēki domā par mani. | | | | 1 | | 2 | 3 | 4 | 5 |
| 46. (80.) | Mani var ātri aizkaitināt. | | | | 1 | | 2 | 3 | 4 | 5 |
| 47. (81.) | Es bieži izdomāju novatoriskas, oriģinālas idejas. | | | | 1 | | 2 | 3 | 4 | 5 |
| 48. (82.) | Man patīk ikviens cilvēks, kuru satieku. | | | | 1 | | 2 | 3 | 4 | 5 |
| 49. (87.) | Mani ir diezgan grūti sadusmot. | | | | 1 | | 2 | 3 | 4 | 5 |
| 50. (88.) | Es bieži atrodu neparastu pielietojumu ierastām lietām. | | | | 1 | | 2 | 3 | 4 | 5 |
| 51. (89.) | Pirms ko daru, es rūpīgi apsveru iespējamās sekas. | | | | 1 | | 2 | 3 | 4 | 5 |
| 52. (90.) | Parasti es sāpīgi uztveru kritiku. | | | | 1 | | 2 | 3 | 4 | 5 |
| 53. (93.) | Varu kļūt nikns pat sīkumu dēļ. | | | | 1 | | 2 | 3 | 4 | 5 |
| 54. (94.) | Diez vai mani varētu nosaukt par radošu cilvēku. | | | | 1 | | 2 | 3 | 4 | 5 |
| 55. (95.) | Es mīlu rūpīgi plānot pat sīkākās aktivitātes un darba lietas. | | | | 1 | | 2 | 3 | 4 | 5 |
| 56. (97.) | Es nejustu sirdsapziņas pārmetumus, ieriebjot kādam, kas man nepatīk. | | | | 1 | | 2 | 3 | 4 | 5 |
| 57. (99.) | Dusmojoties es ātri zaudēju savaldību. | | | | 1 | | 2 | 3 | 4 | 5 |

**Papildus informācija: _________________________________________________________________**

**____________________________________________________________________________________**

**____________________________________________________________________________________**

**____________________________________________________________________________________**

| **N – Neuroticism** = N1 + N2  **N1_new - Anxious-Insecurity**:  7 (14), 14R (27R), 18 (33), 21R (39R), 25 (45), 33 (58), 40 (70), 45 (77), 52 (90).  **N2_new - Irritability:**  46 (80), 49R (87R), 53 (93), 57 (99). | **C – Conscientiousness** = C1 + C2 + C4  **C1 - Orderliness:**  1 (1), 3 (7), 6R (13R), 10 (19).  **C2- Self-discipline:**  13 (26), 17R (32R), 24 (44).  **C4 - Prudence:**  44 (76), 51 (89), 55 (95). |
| --- | --- |
| **E – Extraversion** = E1 + E2 + E3  **E1 - Sociability:**  2 (4), 4R (10R), 8 (16).  **E2 - Joyfulness:**  16 (29), 19 (35), 27 (47).  **E3 - Sensation-Seeking:**  30 (54), 34R (60R), 37 (66), 41 (72). | **A – Agreeableness** = A2 + A3 + A4  **A2 - Gentleness:**  20R (36R), 23R (42R), 28R (48R) + 56R (97R)  **A3 - Obedience:**  31R (55R), 35 (61), 38R (67R).  **A4 - Composure:**  46R (80R), 49 (87), 53R (93R), 57R (99R). |
| **O – Openness to Experience** = O1 + O3 + O4  **O1 - Aesthetic Interests:**  5 (12), 9 (18), 11R (24R).  **O3- Inquisitiveness:**  32 (56), 36 (62), 39 (68), 42 (74).  **O4 - Creativity:**  81, 88, 94R, | **H2 – Greed-Avoidance =**  15 (28), 22 (40), 26R (46R). |
| **M – Lie scale =** 12 (25), 29 (50), 43 (75), 48 (82). |  |
